# DNA binding and lesion recognition by the bacterial interstrand DNA crosslink glycosylase AlkX

**DOI:** 10.1038/s44319-026-00785-6

**Published:** 2026-05-08

**Authors:** Yujuan Cai, Dillon E Kunkle, Marcel D Edinbugh, Noah P Bradley, Eric P Skaar, Brandt F Eichman

**Affiliations:** 1 Department of Biological Sciences, Vanderbilt University, Nashville, TN 37232 USA; 2 Department of Pathology, Microbiology, and Immunology, Vanderbilt University Medical Center, Nashville, TN 37232 USA; 3 Vanderbilt Institute for Infection, Immunology, and Inflammation, Vanderbilt University Medical Center, Nashville, TN 37232 USA; 4 Department of Biology, Fisk University, Nashville, TN 37208 USA; 5 Department of Biochemistry, Vanderbilt University, Nashville, TN 37232 USA; 6 Present Address: AbbVie, North Chicago, IL 60064 USA

**Keywords:** DNA Replication, Recombination & Repair, Structural Biology

## Abstract

Interstrand DNA crosslinks (ICLs) are a highly toxic form of DNA damage. ICL repair in both eukaryotes and bacteria involves unhooking of the two strands by specialized DNA glycosylases. We recently established that the human pathogen *Acinetobacter baumannii* contains an ICL glycosylase (AlkX) that facilitates pathogenesis and protects the bacteria from DNA damage and acid stress. However, the physical basis for glycosylase-catalyzed ICL unhooking is unknown. Here, we describe a crystal structure of AlkX bound to DNA representing a product of the ICL unhooking reaction. Mutational analysis of ICL unhooking in vitro and *A. baumannii* sensitivity to the crosslinking agent mechlorethamine enable the identification of several AlkX motifs critical for ICL repair. We also find that a genetic variant from an antibiotic-resistant strain of the human pathogen *Salmonella enterica* reduces AlkX activity in vitro and increases *A. baumannii* sensitivity to DNA crosslinking. This work provides a structural basis for how bacterial ICL glycosylases recognize and repair DNA adducts and contributes additional evidence that ICL repair is important for fitness of human pathogens.

## Synopsis

A crystal structure of a DNA-bound bacterial DNA glycosylase that repairs toxic interstrand crosslinks reveals several features important for enzymatic activity and fitness of the human pathogen *Acinetobacter baumannii*.

- Crystallographic analysis shows how an interstrand DNA crosslink glycosylase engages DNA.
- Structure-guided mutational analysis reveals conserved DNA binding and active site architectural elements important for growth of the human pathogen *Acinetobacter baumannii*.
- The structure also shows how a naturally selected genetic variant in the animal pathogen *Salmonella* inactivates the enzyme.

## Abstract (web summary)

A crystal structure of a DNA-bound bacterial DNA glycosylase that repairs toxic interstrand crosslinks reveals several features important for enzymatic activity and fitness of the human pathogen *Acinetobacter baumannii*.

## Graphical abstract

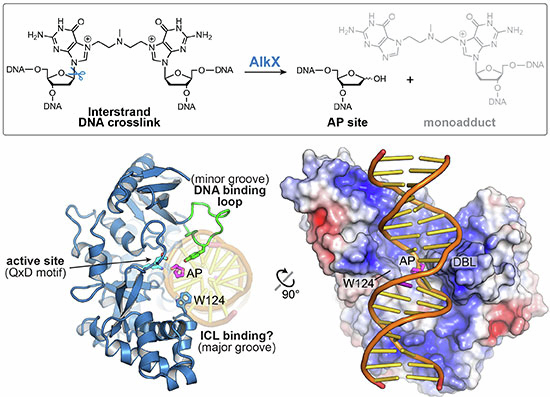

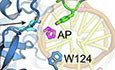

## Introduction

Interstrand DNA crosslinks (ICLs) are a particularly toxic form of DNA damage that covalently tether opposing DNA strands, thus impeding crucial cellular processes such as replication and transcription (Clauson et al, 2013; Noll et al, 2006; Osawa et al, 2011; Schärer, 2005). ICLs are formed from a variety of endogenous cellular metabolites and environmental toxins (Housh et al, 2021; Noll et al, 2006). For example, reactive aldehydes generated from lipid oxidation or alcohol metabolism generate ICLs between opposing guanines (Niedernhofer et al, 2003; Sonohara et al, 2019; Voulgaridou et al, 2011). Similarly, abasic (apurinic/apyrimidinic, AP) sites formed by spontaneous depurination or DNA base excision repair can form ICLs by reacting with exocyclic amino groups on the opposite strand (Amidon and Eichman, 2020; Dutta et al, 2007; Thompson and Cortez, 2020). Microbes and plants also produce crosslinking secondary metabolites (e.g., azinomycin, psoralen, and mitomycin C) (Foulke-Abel et al, 2011; Gates, 1999; Hearst, 1989; Semlow and Walter, 2021). Because of their toxicity, crosslinking agents such as mitomycin C and cisplatin are used as antitumor drugs (Rajski and Williams, 1998; Rycenga and Long, 2018).

**Figure 1:**
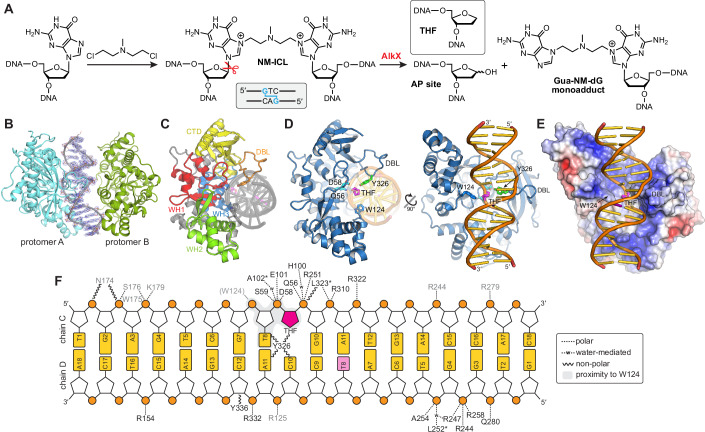
Crystal structure of AlkX bound to DNA. (**A**) NM-ICL formation and hydrolysis. The THF abasic site analog used for structure determination is shown as an inset above. (**B**) TfuAlkX-DNA asymmetric unit, with 2Fo-Fc electron density contoured to 2σ superimposed on the DNA. (**C**) DNA-bound protomer A, colored by domain. (**D**) Orthogonal views of TfuAlkX (blue) bound to THF-DNA (orange/gold). The THF moiety is colored magenta. Active site residues Q56 and D58 are cyan, and Y326 within the DNA-binding loop is green. (**E**) Electrostatic potential surface of TfuAlkX (blue, positive; red, negative), in the same view as the right-hand image in panel (**D**). (**F**) Protein–DNA interactions. Hydrogen bonds and polar interactions are shown as dashed lines, non-polar Van der Waals interactions within 4 Å are shown in wavy lines, and the region in proximity to W124 is shaded gray. Protein residues are colored according to promoter (A, black; B, gray), THF is magenta, and the nucleotide that would be crosslinked by NM is in pink. Asterisks denote main chain contacts.

ICL repair in eukaryotes and prokaryotes involves unhooking the two strands by one of several hydrolytic mechanisms (Bellani et al, 2024; Hodskinson et al, 2020; McVey, 2010; Semlow and Walter, 2021). One unhooking pathway involves DNA glycosylase cleavage of the *N*-glycosidic bond of one of the crosslinked nucleotides, which produces an abasic site on one strand and a monoadduct on the other (Fig. 1A). The abasic site is either replaced by an undamaged nucleotide via the base excision repair (BER) pathway or transiently protected during DNA replication by the HMCES/YedK pathway (Gohil et al, 2023; Krokan and Bjørås, 2013; Mohni et al, 2019; Semlow et al, 2022; Thompson et al, 2019; Thompson and Cortez, 2020). Bacterial ICL glycosylases, which belong to the HTH_42 (PF06224) superfamily of proteins, are prevalent in antibiotic-producing and pathogenic strains (Bradley et al, 2022; Bradley et al, 2020; Mullins et al, 2017b; Wang et al, 2016). In *Streptomyces sahachiroi*, the DNA glycosylase AlkZ unhooks ICLs derived from the bifunctional alkylating agent azinomycin B (AZB) (Bradley et al, 2020; Wang et al, 2016). The *alkZ* gene is embedded within the AZB biosynthetic gene cluster (BGC) and serves as a self-resistance mechanism against AZB toxicity (Chen et al, 2022b; Wang et al, 2016). In *Escherichia coli*, the HTH_42 protein YcaQ hydrolyzes N7-alkylguanosine adducts, including N7-methylguanosine (7mG) monoadducts and ICLs derived from the nitrogen mustard (NM) mechlorethamine (Bradley et al, 2020) (Fig. 1A). Genetic analysis showed that YcaQ initiates a secondary ICL repair pathway that is distinct from the UvrA-initiated nucleotide excision ICL repair pathway (Bradley et al, 2020). YcaQ-mediated ICL repair is dependent on the AP endonuclease, Endo IV, and thus the BER pathway presumably is responsible for processing the AP sites generated from ICL unhooking and excision of the remaining N7-alkylguanine monoadduct, although this has not been firmly established.

AlkZ and YcaQ represent two distinct subgroups within the HTH_42 superfamily (Bradley et al, 2022). Proteins within the AlkZ-like (AZL) subgroup are enriched in BGCs of antibiotic-producing bacteria and are highly specific for DNA adducts formed by their cognate natural product, thus serving an anti-toxin role. The crystal structure of AlkZ revealed a novel architecture in which three winged helix-turn-helix (WH) motifs scaffold a concave, positively-charged DNA-binding surface that contains a highly conserved catalytic QxQ motif (Mullins et al, 2017b). Outside of this motif, AZLs have relatively low sequence similarity, consistent with their varied substrate specificities (Bradley et al, 2022; Chen et al, 2022a; Mullins et al, 2017b). In contrast, the YcaQ-like (YQL) proteins are not found in BGCs, are prevalent in pathogenic bacteria, and share high sequence conservation, including a QxD catalytic motif (Bradley et al, 2022; Bradley et al, 2020). Thus, whereas AZLs have evolved specificity for a particular secondary metabolite in an antibiotic producer, YQLs unhook a variety of ICLs and thus likely provide a more general defense against endogenous and exogenous genotoxins. Although the crystal structure of AlkZ has been determined, the structural features of the YQL subfamily are unknown.

We recently characterized an *Acinetobacter baumannii* YQL ortholog, which we named AlkX, as an ICL-DNA glycosylase that protects the bacteria against mechlorethamine toxicity (Kunkle et al, 2024). *A. baumannii* is a highly antibiotic-resistant Gram-negative nosocomial pathogen that is a leading cause of antibiotic-resistant attributable deaths globally (Collaborators, 2024). *A. baumannii* strains that have evolved resistance to last-resort antibiotics are commonly isolated from patients, leading the WHO to identify *A. baumannii* as a critical priority pathogen, indicating the need for the development of new antibacterials to treat these drug-resistant infections (Sati et al, 2025). AlkX promotes *A. baumannii* colonization of the lungs and dissemination to distal tissues during pneumonia. In addition to protecting cells against ICL agents, *alkX* is induced by and protects against acid stress—a condition the bacteria encounter while interacting with immune cells in the host—although the relationship between the acid stress and DNA damage responses of AlkX are unclear (Kunkle et al, 2024).

Despite the importance of AlkX for *A. baumannii* fitness and of AlkX and other YQL DNA glycosylases for ICL repair in prokaryotes, the mechanism by which they unhook ICLs is poorly understood. Moreover, we still do not understand the molecular basis for differences between YQL and AZL subclades of these enzymes. To fill these gaps in knowledge, we determined a crystal structure of AlkX bound to DNA containing an abasic site, representing one-half of the product of the glycosylase reaction (Fig. 1A). The structure revealed DNA binding motifs that clamp the damage site from both major and minor grooves. Mutational analysis of these motifs confirmed their importance for substrate binding, ICL unhooking, and *A. baumannii* fitness. We also identified a critical structural feature of the active site that would be perturbed in a putative AlkX variant found in an antibiotic-resistant strain of another human pathogen, *Salmonella enterica* (Jones-Dias et al, 2016), providing additional evidence for the importance of ICL unhooking activity by this family of proteins in pathogenic bacteria. Comparison of the AlkX and AlkZ structures provides a basis for the substrate differences in the YQL and AZL subfamilies. Together, these results provide molecular insight into the recognition and repair of ICLs by DNA glycosylases important for viability and pathogenesis in bacteria.

## Results and discussion

### Crystal structure of AlkX bound to DNA

**Figure EV1:**
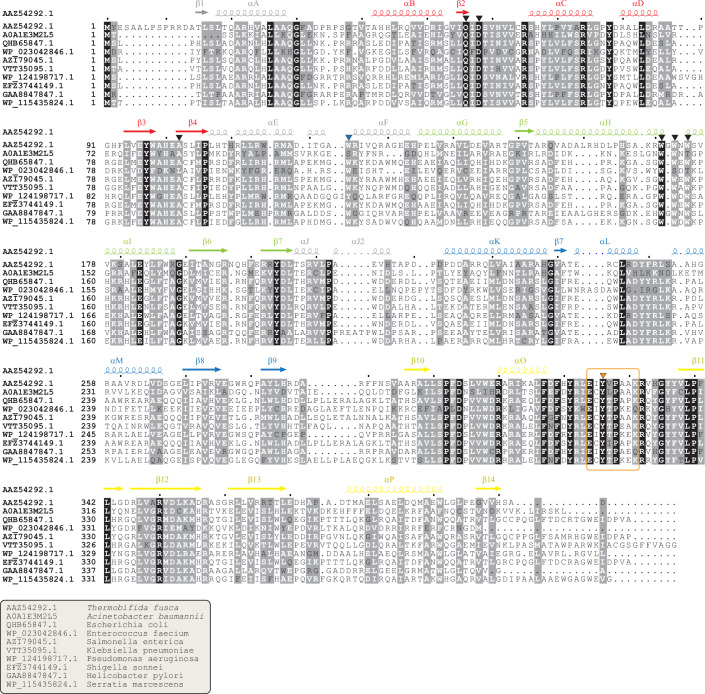
Sequence alignment of YQL/AlkX proteins. Secondary structure elements are colored by domain as in Fig. 1C. The DBL is highlighted with an orange box. Triangles denote residues mutated in this study. The orange and blue triangles designate the DBL tyrosine and the putative ICL recognition motif, respectively. The organisms corresponding to the sequence accession numbers are shown at the bottom of the figure.

**Figure EV2:**
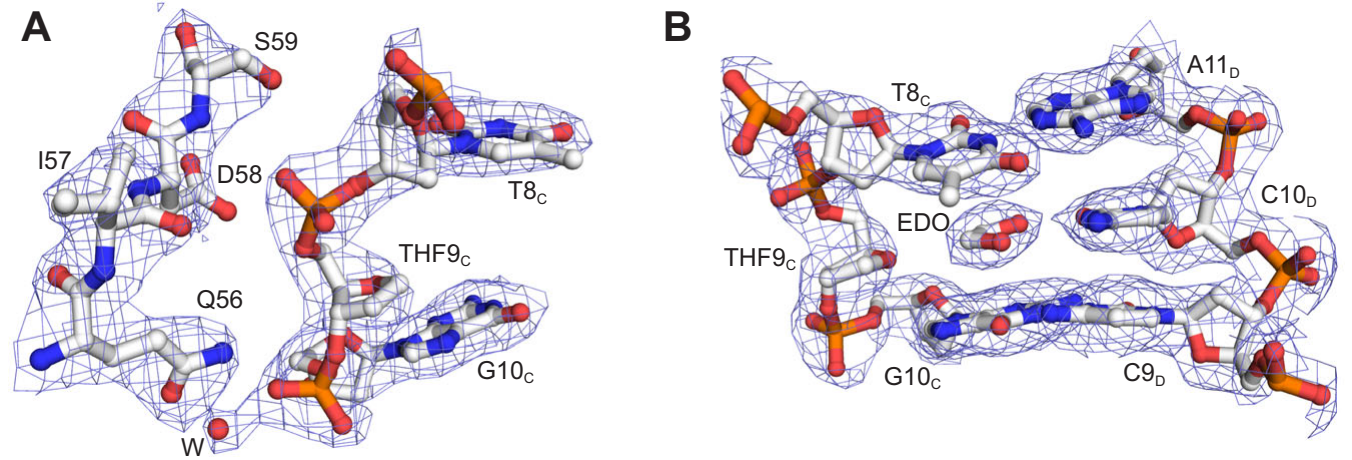
Electron density. Interaction of the THF region with the active site (**A**) and the opposite DNA strand (**B**). 2Fo-Fc electron density contoured to 1σ is superimposed.

After screening several YQL/AlkX orthologs and oligonucleotides, we obtained diffraction-quality crystals of the *Thermobifida fusca* enzyme (TfuAlkX) bound to DNA. TfuAlkX unhooks NM-ICLs and excises 7mG monoadducts (Bradley et al, 2022), and shares 30% sequence identity and 47% similarity to *A. baumannii* (Aba) AlkX and 34% identity and 50% similarity to *E. coli* YcaQ (Fig. EV1). TfuAlkX was crystallized with an 18-mer DNA oligonucleotide containing a centrally located tetrahydrofuran (THF) abasic site analog, which represents one-half of the ICL unhooking product since the DNA does not contain a guanine-NM-dG monoadduct on the opposite strand (Fig. 1A). The structure was determined to 2.6 Å resolution by single-wavelength anomalous dispersion (SAD) from selenomethionine-substituted protein and refined to a crystallographic residual (*R*-factor) of 20.7% (R_free_ = 25.6%) (Fig. EV2 and Table EV1). The asymmetric unit contained two protomers and one DNA duplex, with only one protomer engaged with the DNA (Fig. 1B).

**Figure EV3:**
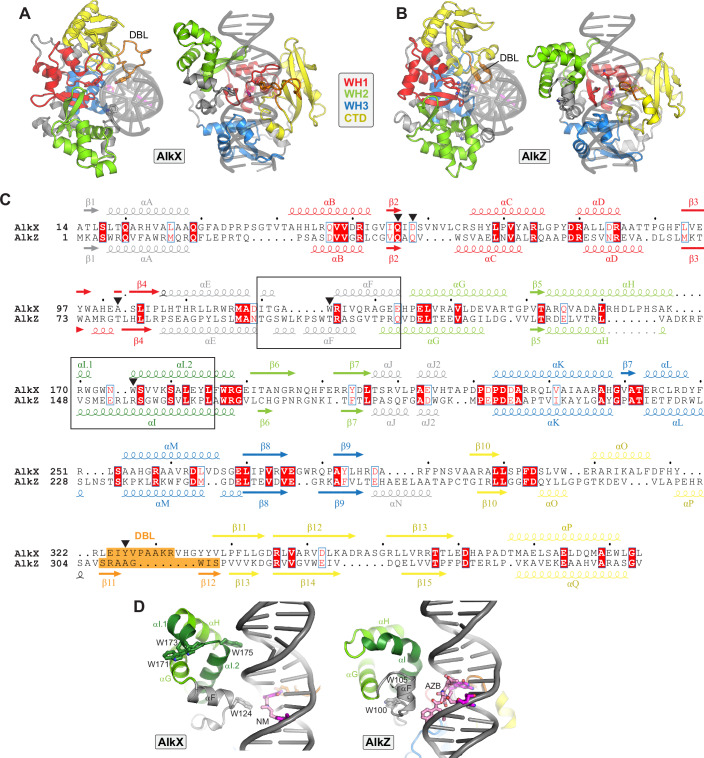
Comparison of AlkX and AlkZ structures. (**A**) Crystal structure of TfuAlkX bound to THF-DNA. The protein is colored by domain (WH1 red, WH2 green, WH3 blue, C-terminal domain yellow, DBL orange). (**B**) Crystal structure of *Streptomyces sahachiroi* (Ssa) AlkZ (PDB ID 5UUJ) docked against THF-DNA from the TfuAlkX structure. (**C**) Sequence alignment of TfuAlkX and SsaAlkZ. Putative lesion-sensing regions αF and αI are boxed. Residues important for ICL unhooking in AlkX are marked with black triangles. (**D**) Comparison of αF and WH2 regions of AlkX (left) and AlkZ (right). DNA models of NM- and azinomycin B (AZB)-ICLs are based on the DNA in the TfuAlkX structure.

TfuAlkX adopts a 3xWH/β-barrel architecture similar to that of *Streptomyces sahachiroi* AlkZ (Figs. 1C and EV3). The RMSD between TfuAlkX and AlkZ is 3.99 Å for 269 C_α_ atoms, highlighting the conservation of the HTH_42 fold. Consistent with our previous prediction for AlkZ (Mullins et al, 2017b), TfuAlkX binds the DNA against its concave, positively-charged surface, with the THF moiety close to the catalytic QxD motif (Figs. 1D,E and EV2). Although *N*7-methylguanine (7mGua) nucleobase was included in the crystallization condition to stabilize the enzyme-product complex (Mullins et al, 2015), it was not evident in the electron density. Instead, a molecule of ethylene glycol from the crystallization condition was observed to fill the nucleobase void beside the THF abasic site (Fig. EV2), suggesting that the excised nucleobase does not remain bound to AlkX. The THF (lesion) site is clamped between an extensive DNA-binding loop (DBL) from the β-sheet subdomain in the minor groove and a tryptophan side chain (W124) from the region between WH1 and WH2 in the major groove (Fig. 1D–F). Thus, the resolution and quality of the electron density in the structure reveal, for the first time, several critical interactions between a bacterial ICL glycosylase and damaged DNA that we describe in more detail below.

### The DNA-binding loop is important for AlkX function

**Figure 2:**
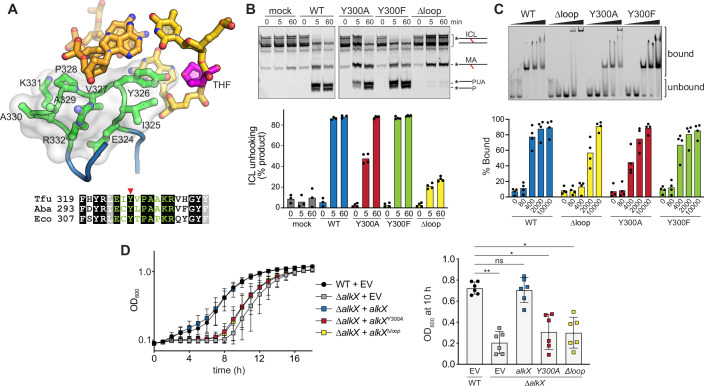
The DNA-binding loop (DBL) is important for AlkX function. (**A**) Structure of the DBL (green) in contact with the DNA minor groove at the lesion site (magenta THF). The solvent accessible surface of the DBL is shown in transparent white. The sequence alignment of the DBL between AlkX/YQL orthologs from *Thermobifida fusca* (Tfu), *Acinetobacter baumannii* (Aba), and *Escherichia coli* (Eco) is shown below. The conserved tyrosine is highlighted with a red triangle. (**B**) NM-ICL unhooking by AbaAlkX wild-type (WT) and mutants. A representative denaturing polyacrylamide gel shows conversion of NM-ICL substrate to a monoadduct (MA) strand and alkali-cleaved AP-strand products. PUA, 3′-phospho-α,β-unsaturated aldehyde; P, 3′-phosphate; mock, no enzyme. Quantification shows the mean of 3-4 independent experiments. (**C**) Electrophoretic mobility shift assay (EMSA) of AlkX mutants binding to 2F-NM_8_-ICL DNA (Appendix Fig. S1). Quantification of four replicate experiments is shown below. (**D**) Growth curves of *A. baumannii* WT and Δ*alkX* strains harboring either empty vector (EV) or indicated *alkX* expression vectors in the presence of 33 µM mechlorethamine. Data represent the mean ± SD of six biological replicates performed in technical triplicate. The comparison of strain viability harboring different mutants is shown on the right with the OD_600_ values at 10 h. Data represent the mean ± SD; each dot represents an individual biological replicate performed in technical triplicate. *P = 0.0260, **P = 0.0026, ns (not significant) as determined by Dunn’s multiple comparisons test. Source data are available online for this figure.

**Figure EV4:**
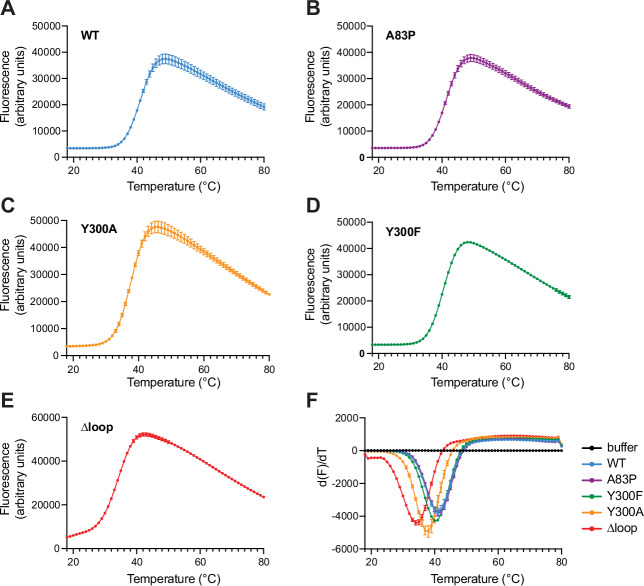
Thermostability of AlkX mutants. (**A**–**E**) Differential scanning fluorescence thermal denaturation profiles for AlkX WT (**A**), A83P (**B**), Y300A (**C**), Y300F (**D**), and Δloop (**E**). Data represent the mean ± SD (*n* = 3). Samples contained 10 μM protein, 20 mM Tris, pH 8.0, 150 mM NaCl, 1 mM TCEP, 0.1 mM EDTA, and 0.5× SYPRO Orange and measurements were carried out as previously described (Dorival et al, 2025). (**F**) First derivatives of the thermal denaturation data in panels (**A**–**E**). The minima signify the melting temperatures (*T*_m_). Data represent the mean ± SD (*n* = 3).

Perhaps the most striking feature of the TfuAlkX structure is the DBL protruding into the minor groove at the lesion site (Fig. 1D,E). The DBL is located within the β-sheet subdomain and contains 18 residues (F319–Y336), nine of which (E324–R332) adopt a cradle for the phosphoribose backbone of the strand opposite the THF (Fig. 2A). At the tip of the loop, a conserved tyrosine (Y326) partially fills the abasic site void and forms van der Waals contacts with the THF and the nucleobases around the THF. The DBL is in a similar position as the β11/12 motif of AlkZ that we previously showed to be essential for glycosylase activity (Fig. EV3) (Mullins et al, 2014). To determine the importance of the DBL in AlkX function, we generated mutants within the corresponding region in AbaAlkX and examined NM-ICL unhooking activity in vitro (Fig. 2B; Appendix Fig. S1). In this assay, AlkX is incubated with a Cy5-labeled NM-ICL substrate, which generates a guanine-NM-dG monoadduct (MA) strand and an AP strand (Fig. 1A) that can be separated by electrophoresis after alkali nicking of the AP strand (Bradley et al, 2020). ICL unhooking was severely curtailed in a “Δloop” mutant in which AbaAlkX loop residues E298–R306 (corresponding to E324–R332 in TfuAlkX) were replaced with GSSG (Fig. 2B). Similarly, ICL unhooking was reduced by alanine substitution of the tyrosine side chain (Y300A). We verified that the reduction in activity by the mutants was not the result of reduced protein thermostability (Fig. EV4). Phenylalanine substitution of Y300 had no effect on ICL unhooking, indicating that the hydroxyl group is not important for catalysis. The corresponding mutants in TfuAlkX—Δloop (E324–R332 > GSSG), Y326A, and Y326F—showed the same effect as those in AbaAlkX (Appendix Fig. S2).

We also probed the effect of the mutations on AbaAlkX binding to DNA containing a stable NM-ICL constructed of an 8-atom NM analog, which we previously showed to be a substrate for *E. coli* YcaQ (Bradley et al, 2020), and C2′-fluorinated guanines to prevent glycosylase cleavage (Fig. 2C; Appendix Fig. S1). Similar to the ICL unhooking results, DNA binding was severely curtailed by the Δloop mutant, reduced by Y300A, and unaffected by Y300F, indicating that the tyrosine contributes sterically to DNA binding by the DBL. Consistent with the importance of the DBL in DNA binding, electron density for the DBL was not observed in the protomer not engaged with the DNA, suggesting that this motif is disordered in the absence of DNA.

Given the effect of Δloop and Y300A mutants on ICL unhooking and DNA binding, we probed the effect of these mutations in vivo by measuring their ability to complement an *A. baumannii* Δ*alkX* mutant strain grown in the presence of the DNA crosslinker mechlorethamine. Consistent with the in vitro results, the DBL mutants increased the sensitivity of *A. baumannii* DNA crosslinking, as monitored by cell growth (Fig. 2D; Appendix Fig. S1). The Δ*alkX* strain showed greater sensitivity compared to WT, as judged by delayed growth. This sensitivity was complemented by expression of wild-type *alkX*. However, expression of either *Y300A* or Δ*loop alkX* mutant alleles failed to complement the loss of *alkX*. Thus, cells harboring these mutants exhibit greater mechlorethamine sensitivity.

Collectively, these findings have uncovered a previously unknown region of AlkX that is required for ICL repair. Based on the extremely high conservation of the DBL across YQL/AlkX proteins (Fig. EV1), it stands to reason that these proteins will process ICLs in the same manner. We speculate that in addition to stabilizing the DNA in an orientation that promotes catalysis, the DBL contributes to ICL detection by recognizing crosslink-induced distortions to the DNA. This assertion is supported by the extensive contacts between the DBL and the minor groove (Fig. 2A) and the unique and conserved DBLs within each YQL and AZL subfamily, each of which has distinct specificities (Bradley et al, 2022; Bradley et al, 2020; Mullins et al, 2017b).

### The AlkX active site contains a genetic variant in *S. enterica* that impairs ICL repair

**Figure 3:**
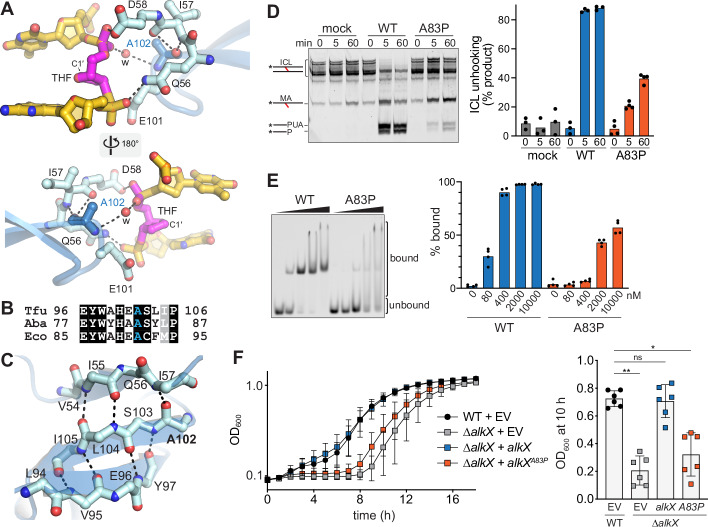
The AlkX active site contains a genetic variant in *S. enterica* that impairs ICL repair. (**A**) Crystal structure of the active site, showing the proximity of A102 main chain to the catalytic QxD motif. Hydrogen bonds are shown as dashed lines, and a bridging water is shown as a red sphere. (**B**) Sequence alignment of this region in AlkX**/**YQL proteins from *T. fusca* (Tfu), *A. baumannii* (Aba), and *E. coli* (Eco). (**C**) The A102 main chain is linked to the QxD motif within a β-sheet in the WH1 domain. Only main chain atoms are shown. (**D**) Representative gel and quantification of ICL unhooking activity of AbaAlkX A83P. Bars denote the mean from 3 to 4 replicates. The gel (mock and WT) is the same as that shown in Fig. 2B and is duplicated here for comparison to A83P. (**E**) Representative EMSA of AbaAlkX A83P binding to 2F-NM_8_-ICL, with quantification showing the mean from four independent measurements. (**F**) Growth curves of *A. baumannii* WT and Δ*alkX* strains harboring empty vector (EV) or indicated *alkX* expression vectors in the presence of 33 µM mechlorethamine. Data represent the mean ± SD of six biological replicates performed in technical triplicate. Comparison of strain viability harboring *alkX* WT and A83P is shown on the right with the OD_600_ values at 10 h. Data represent the mean ± SD; each dot represents an individual biological replicate. *P = 0.0269, **P = 0.0033, ns (not significant) as determined by Dunn’s multiple comparisons test. Source data are available online for this figure.

The glutamine and aspartate residues within the conserved catalytic QxD motif are essential for base excision in *E. coli* YcaQ and AbaAlkX (Bradley et al, 2020; Kunkle et al, 2024). We postulate that one or both of these residues stabilize the position of the catalytic water for attack of the lesion C1′ (Mullins et al, 2019; Mullins et al, 2017b). In the TfuAlkX crystal structure, the Q56 and D58 side chains of the QxD motif form hydrogen bonds with the phosphate groups 5′ and 3′ to the THF nucleotide (Fig. 3A). As a result, neither side chain is close enough to the C1′ carbon to catalyze hydrolysis, and thus the structure suggests that the enzyme and/or DNA undergo a small conformational change after cleavage of the N-glycosidic bond. The phosphate interactions impart a deformation in the THF backbone such that the THF ring is rotated 90° relative to the position of the deoxyribose rings in duplex DNA, with the C1′ carbon facing the DNA duplex (Fig. 3A). This position, together with the lack of empty space between the THF and the protein, is consistent with a non-base-flipping catalytic mechanism expected for an ICL glycosylase (Mullins et al, 2019). Although the structure revealed a glutamate (E101) side chain close to the C1′ carbon, alanine substitution of this residue in TfuAlkX (E101A) did not affect ICL unhooking activity (Appendix Fig. S2), and thus we conclude that it likely does not participate in catalysis. This was not unexpected since this residue, while conserved in most AlkX/YQL orthologs, is an alanine in AbaAlkX (Figs. 3B and EV1).

The structure revealed an important element of the active site that likely stabilizes the positions of both the QxD motif and the DNA lesion. At the end of the β-hairpin in WH1 (the “wing” of the winged-helix), the main chain atoms of residue A102 form a direct hydrogen bond with the main chain of I57 within the QxD (QID) motif and a water-mediated hydrogen bond with the THF phosphate (Fig. 3A,C). Highly conserved in AlkX/YQL proteins (Fig. EV1), A102 is also involved in a three-stranded β-sheet and thus is an important structural feature that imparts stability to the active site (Fig. 3C). Interestingly, a corresponding alanine in a putative YQL ortholog in *Salmonella enterica* is mutated to a proline in a single-nucleotide variant (A89P) found in a tetracycline-resistant strain of *S. enterica* serotype Enteritidis (Jones-Dias et al, 2016). *S. enterica* is a dangerous human pathogen that causes gastrointestinal infections related to the consumption of contaminated animal products (Foley et al, 2013). Based on the importance of this alanine to the structure of the AlkX active site, we hypothesized that the *S. enterica* A89P mutation likely alters the position of the catalytic motif to consequently impair ICL unhooking activity. Consistent with this assertion, we found that proline substitution of the corresponding alanine in AbaAlkX (A83P) dramatically reduced ICL unhooking and ICL DNA binding in vitro (Fig. 3D,E; Appendix Fig. S1) without destabilizing the thermostability of the protein (Fig. EV4). In vivo, the AbaAlkX A83P mutation sensitized the Δ*alkX A. baumannii* strain to mechlorethamine to the same extent as the DBL and QxD mutations, the latter of which we tested previously (Figs. 2D and 3F; Appendix Fig. S1) (Kunkle et al, 2024). We confirmed that a TfuAlkX A102P mutant also impaired ICL unhooking activity (Appendix Fig. S2). Thus, the WH1 motif is a conserved feature in AlkX orthologs that stabilizes the catalytic QxD motif for ICL unhooking. Furthermore, our structure of an AlkX/YQL ICL glycosylase from a pathogenic bacterium has provided a possible mechanistic explanation for how a genetic variant found in an antibiotic-resistant strain of *S. enterica* inactivates the glycosylase.

### Identification of a putative ICL sensor in AlkX

**Figure 4:**
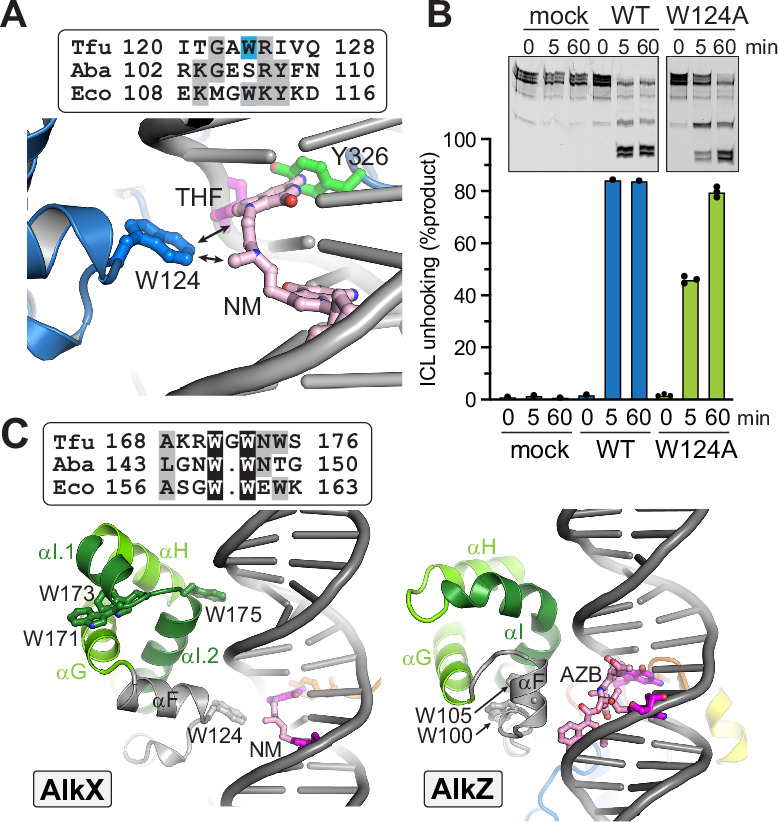
Identification of a putative ICL sensor in AlkX. (**A**) Model of TfuAlkX bound to the full product of the NM-ICL unhooking reaction, based on the TfuAlkX/THF-DNA structure. The Gua-NM-dG monoadduct is shown in pink, and the THF from the crystal structure is magenta. A sequence alignment of the αF region in YQLs from *T. fusca* (Tfu), *A. baumannii* (Aba), and *E. coli* (Eco) is shown above. Double-headed arrows denote potential steric interactions between the protein and ICL. (**B**) ICL unhooking activity of TfuAlkX W124A. Data represent the mean from three independent replicates. (**C**) Comparison of αF (gray) and WH2 (green) regions of AlkX (left) and AlkZ (right). DNA models of NM- and azinomycin B (AZB)-ICLs are based on the DNA in the TfuAlkX structure. The NM and AZB moieties are pink, crosslinked nucleases magenta, and DNA binding loops orange. A sequence alignment of the αI region in AlkX orthologs is shown above. Source data are available online for this figure.

On the opposite side of the DNA duplex from the DBL, W124 penetrates the major groove at the lesion site (Figs. 1D,E and 4A). W124 is located at the N-terminal end of helix αF, in the region connecting WH1 and WH2 (Fig. EV3). The W124 side chain does not make any DNA contacts in the structure but resides ~5 Å from the THF and the nucleotide 5′ to the THF (Fig. 1F). However, modeling a NM onto the DNA suggests that W124 would contact the crosslink (Fig. 4A). Indeed, mutation of W124 to alanine in TfuAlkX reduced ICL unhooking relative to the wild-type protein, although to a lesser extent than that of the DBL Y326A mutant on the other side of the DNA or the A102P mutant in the active site (Fig. 4B; Appendix Fig. S2). Nonetheless, the position of this residue and its reduction in NM-ICL activity suggests that it plays a role in sensing the crosslink through direct van der Waals contacts or a cation-π interaction involving the indole ring and the positively-charged guanine-N7 nitrogen. Consistent with this, this αF region is relatively well conserved among YQL proteins but divergent among AZLs, which have evolved specificities for DNA adducts of a particular natural product (Bradley et al, 2022; Bradley et al, 2020; Chen et al, 2022a). It is not surprising, therefore, that the conformations of the αF regions in the TfuAlkX and *Streptomyces sahachiroi* AlkZ structures are different (Fig. 4C). This difference, however, may simply reflect a DNA-bound (AlkX) versus DNA-free (AlkZ) conformation. Interestingly, although the lesion-sensing tryptophan in TfuAlkX is highly conserved among AlkX/YQL proteins, it is a serine in AbaAlkX (Figs. 4A and EV1). Thus, it is possible that this motif discriminates among ICLs or minor groove lesions in an organism-specific manner.

We previously predicted the αI helix within WH2 to serve as a lesion recognition motif in the Streptomyces AZL proteins AlkZ, TxnU2/4, and LldU1/5, based on its high sequence divergence and predicted interaction with the lesion site in models constructed from the AlkZ structure (Chen et al, 2022a; Mullins et al, 2017b). In contrast, in TfuAlkX, WH2 contacts the DNA one-half turn up the duplex from the αF region (Fig. 4C) and therefore does not contact the lesion. Helix αI, which is highly kinked in AlkZ, is separated into two shorter α-helices and connected with a triple tryptophan loop (WGWNW) in TfuAlkX. W171 and W173 face into the WH2 core while W175 points toward the DNA minor groove (Figs. 4C and EV3). Consistent with the structure, substitution of W175 with alanine only modestly reduced ICL unhooking by TfuAlkX, and a W173A mutant had no effect (Appendix Fig. S3). W171 and W173, but not W175, are conserved in AbaAlkX, and mutation of these residues had no effect on ICL unhooking in AbaAlkX (Appendix Fig. S3). Thus, we conclude that the WH2 region does not play a role in ICL unhooking in the YQL/AlkX subfamily. It remains to be determined if this region serves as an ICL recognition motif in the AZLs. Modeling AlkZ against an AZB-ICL using the DNA from the TfuAlkX structure does not place helix αI in proximity to the crosslink (Fig. 4C). However, the current structure represents a relaxed (non-crosslinked) DNA conformation, and thus it is difficult to approximate the proximity of the αF and αI helices to the lesion in a true ICL substrate. Nevertheless, the structure and supporting mutational analysis strongly suggest that YQL enzymes recognize guanine-N7-linked (major groove) ICLs by making direct contacts to the ICL through their αF and/or αI motifs, and by sensing ICL-induced distortions to the DNA through extensive interactions between the DBL and the minor groove. Future investigation into these regions is needed to discern their effects on the substrate specificities of both YQL and AZL glycosylases.

A putative ICL sensor in AlkX that differs between TfuAlkX and AbaAlkX raises the possibility that AlkX/YQL proteins have evolved to serve different DNA repair processes in individual species. This hypothesis is supported by the observation that *E. coli ycaQ* transcription is under the regulation of a σ^70^-dependent promoter, and this architecture is conserved in *Salmonella enterica*, suggesting that the YQL genes in these organisms are constitutively expressed (Bradley et al, 2020). However, *alkX* expression in *A. baumannii* is induced in response to mechlorethamine treatment, and treatment of *P. aeruginosa* with a quorum-sensing inhibitor induces expression of its *alkX* homolog (Tan et al, 2014). Collectively, these findings suggest the possibility that distinct organisms have evolved alterations in YQL protein structures as well as species-specific regulatory mechanisms to control the expression of YQL proteins that serve specific functions in distinct niches.

To our knowledge, the present work reports the first structural information for how a bacterial ICL glycosylase engages DNA and achieves specificity for an ICL. Our AlkX-DNA structure revealed several conserved motifs that we found to be important for ICL unhooking and recognition, mutation of which led to increases in *A. baumannii* sensitivity to DNA crosslinking. Given the widespread distribution of AlkX/YQL ICL glycosylases in pathogenic bacteria, including *A. baumannii*, *Salmonella enterica*, *Listeria monocytogenes*, and *Pseudomonas aeruginosa*, it will be important to determine the role of these enzymes in pathogenesis and to define their endogenous substrates.

While DNA glycosylases traditionally have been regarded as a means of repairing nucleobases containing small modifications from oxidation, deamination, and alkylation as a component of the base excision repair (BER) pathway, they are now established as a means of repairing bulky adducts and ICLs in both prokaryotes and eukaryotes (Baiken et al, 2020; Bradley et al, 2022; Bradley et al, 2020; Kothandapani and Patrick, 2013; Mullins et al, 2019; Mullins et al, 2017a; Mullins et al, 2015; Semlow et al, 2016; Wang et al, 2016; Xu et al, 2012). The bacterial ICL glycosylases characterized to date (AlkZ, AlkX/YcaQ) seem to be specific for compounds that link guanines through the N7 nitrogen (Bradley et al, 2020; Gates et al, 2004; Mullins et al, 2017b; Wang et al, 2016), and related AZL glycosylases recognize N7-alkylated monoadducts (Bradley et al, 2022; Chen et al, 2022a). In contrast, the eukaryotic DNA glycosylase NEIL3 (Endonuclease VIII-like 3) repairs AP- and psoralen-ICLs as an alternative to the Fanconi Anemia (FA) pathway and to process double-strand breaks in FA-dependent repair of mitomycin C- and cisplatin-ICLs (Li et al, 2020; Li et al, 2022; Oswalt and Eichman, 2024; Semlow et al, 2016). Unlike the bacterial ICL glycosylases, NEIL3 cannot unhook ICLs from double-stranded DNA; it requires a double/single-stranded (splayed arm) junction that would be present at replication forks, consistent with its cellular recruitment to ICLs present at converged forks (Imani Nejad et al, 2020; Semlow et al, 2016; Wu et al, 2019). Moreover, the NEIL3 glycosylase domain adopts an entirely different fold than AlkX and AlkZ (Huskova et al, 2022; Liu et al, 2013) and contains DNA-binding domains outside of the glycosylase domain (Huskova et al, 2022; Imani Nejad et al, 2020; Rodriguez et al, 2020). Thus, the bacterial and eukaryotic glycosylases have evolved distinct architectures and substrate specificities, although it remains to be determined if there are similarities in how they recognize and unhook their respective crosslinks.

## Methods

**Table:** Reagents and tools table

Reagent/resource	Reference or source	Identifier or catalog number
**Experimental models**		
*E. coli* DH5α	Lab stock	N/A
*A. baumannii* 17978VU	Lab stock	N/A
*A. baumannii* Δ*alkX*	Kunkle et al (2024) Proc Natl Acad Sci, USA	Prof. Dr. Eric P. Skaar, Vanderbilt University, United States
*E. coli* Tuner (DE3)	Lab stock	N/A
**Recombinant DNA**		
pWH1266	Hunger et al (1990) Gene	Prof. Dr. Wolfgang Hillen, Friedrich-Alexander Universität Erlangen-Nümberg, Germany
pWH1266-*alkX*	Kunkle et al, (2024) Proc Natl Acad Sci USA	Prof. Dr. Eric P. Skaar, Vanderbilt University, United States
pWH1266-*alkX*^*A83P*^	This study	N/A
pWH1266-*alkX*^*Y300A*^	This study	N/A
pWH1266-*alkX*^*Δloop*^	This study	N/A
pBG102	Vanderbilt Center for Structural Biology	N/A
**Oligonucleotides and other sequence-based reagents**		
Biochemical experiments		
TGAGTCGT(THF)GATGACCAC	Integrated DNA Technologies	top strand of THF-DNA for crystallization
GTGGTCATCCACGACTCA	Integrated DNA Technologies	bottom strand of THF-DNA for crystallization
Cy5-TTTATTTTTATTTGACTTTTATTTTT	Integrated DNA Technologies	top strand for NM-ICL preparation
FAM- AAAAATAAAAGTCAAATAAAAATAAA	Integrated DNA Technologies	bottom strand for NM-ICL preparation
Cy5-TTTATTTTTATTTG^F^ACTTTTATTTTT	Integrated DNA Technologies	top strand for NM-ICL preparation in EMSA
FAM- AAAAATAAAAG^F^TCAAATAAAAATAAA	Integrated DNA Technologies	bottom strand for NM-ICL preparation in EMSA
Primers		
TAGGCTTGGTTATGCCGGTACTG	Integrated DNA Technologies	pWH1266_seq_F
GGAAGGAGCTGACTGGGTTGA	Integrated DNA Technologies	pWH1266_seq_R
GTATCATGCGCCGTCTTATCTTCCTATGAAAG	Integrated DNA Technologies	pWH1266-AlkXA83P_F
CAATATTCGAAAATCTGCCGTTCACGAACCAAAC	Integrated DNA Technologies	pWH1266-AlkXA83P_R
GATCGAATGCGCGTTACCAGCGGCCAAAC	Integrated DNA Technologies	pWH1266-AlkXY300A_F
CGATAATCAAATTCAAATAAGGAAGTTAAACGGTC	Integrated DNA Technologies	pWH1266-AlkXY300A_R
GCGACCACACCCGTCCTGTGGATCCTAAAGGTTTAGGTGAGTAAAG	Integrated DNA Technologies	pWH1266-AlkX_Δloop_F
AAGGCTCTCAAGGGCATCGGTCGACTTAAAGCTTGCTGCGAATG	Integrated DNA Technologies	pWH1266-AlkX_Δloop_R
CATGCACCGAGCTATCTGCCGATGAAGGAC	Integrated DNA Technologies	AbaAlkXA83P_F
CAGATAGCTCGGTGCATGATACCAGTACTC	Integrated DNA Technologies	AbaAlkXA83P_R
GTATTGAGTGCGCTCTGCCAGCTGCCAAAC	Integrated DNA Technologies	AbaAlkXY300A_F
CAGCTGGCAGAGCGCACTCAATACGATAGTC	Integrated DNA Technologies	AbaAlkXY300A_R
GTATTGAGTGCTTTCTGCCAGCTGCCAAAC	Integrated DNA Technologies	AbaAlkXY300F_F
CAGCTGGCAGAAAGCACTCAATACGATAGTC	Integrated DNA Technologies	AbaAlkXY300F_R
CTATCGTATTGGCAGTAGTGGGGTCTTTGGCTACTTCTGCCTTCC	Integrated DNA Technologies	AbaAlkXΔloop_F
GCCAAAGACCCCACTACTGCCAATACGATAGTCAAACTCAAACAAGC	Integrated DNA Technologies	AbaAlkXΔloop_R
GCACGAACCGAGCCTGATTCCGCTGCACAC	Integrated DNA Technologies	TfuAlkXA102P_F
CGGAATCAGGCTCGGTTCGTGCGCCCAATACTC	Integrated DNA Technologies	TfuAlkXA102P_R
CCGTCTGGAAATTGCTGTGCCGGCGGCGAAAC	Integrated DNA Technologies	TfuAlkXY326A_F
GTTTCGCCGCCGGCACAGCAATTTCCAGACG	Integrated DNA Technologies	TfuAlkXY326A_R
TCTGGAAATTTTTGTGCCGGCGG	Integrated DNA Technologies	TfuAlkXY326F_F
CGGTAGTGAAAATCGAACAGC	Integrated DNA Technologies	TfuAlkXY326F_R
GCTGTTCGATTTTCACTACCGTCTGGGCAGTAGTGGGGTGCACG	Integrated DNA Technologies	TfuAlkXΔloop_F
CGGCAGAACATAGTAACCGTGCACCCCACTACTGCCCAGACGGT	Integrated DNA Technologies	TfuAlkXΔloop_R
**Chemicals, Enzymes and other reagents**		
*N*^*1*^*,N*^*2*^-bis(2-chloroethyl)-*N1,N2*-dimethylethane-1,2-diamine	Bradley et al (2022) Nucleic Acid Res	Prof. Dr. Brandt F. Eichman, Vanderbilt University, United States
CloneAmp HiFi PCR Premix	Takara Bio	Cat.#639298
KAPA HiFi HotStart ReadyMix	Roche Diagnostics	Cat.#07958927001
DpnI	New England Biolabs	Cat.#R0176
SalI	New England Biolabs	Cat.#R0138
BamHI	New England Biolabs	Cat.#R0136
NEBuilder HiFi DNA Assembly	New England Biolabs	Cat.#E2621
Novex 15% TBE-Urea Gel	Thermo Fisher	Cat.#EC6885
NuPAGE 4-12% Bis-Tris Gel	Thermo Fisher	Cat.#NP0329BOX
40% Acrylamide/Bis Solution, 19:1	Bio-Rad	Cat.#1610144
Acrylamide	Sigma-Aldrich	Cat.#A3553
*N*,*N*’-Methylenebisacrylamide	Sigma-Aldrich	Cat.#M7279
Orange G	Sigma-Aldrich	Cat.#O1625
Kanamycin sulfate	Sigma-Aldrich	Cat.#K1377
LB Broth	Fisher Scientific	Cat.#BP9723-2
Sodium chloride	Thermo Scientific	Cat.#42429-0050
TCEP-HCl	Hampton Research	Cat.#HR2-801
IPTG	Sigma-Aldrich	Cat.#I6758
PMSF	AmericanBio	Cat.#AB01620
Glycerol	Fisher Scientific	Cat.#G33-4
L-Selenomethionine	Sigma-Aldrich	Cat.#561505
L-Phenylalanine	Thermo Scientific	Cat.#A13238
L-Lysine	Sigma-Aldrich	Cat.#L-5626
L-Threonine	Thermo Scientific	Cat.#138931000
L-Leucine	Thermo Scientific	Cat.#A12311
L-Isoleucine	Thermo Scientific	Cat.#166171000
L-Valine	Thermo Scientific	Cat.#A12720
Trizma base	Sigma-Aldrich	Cat.#T6066
Formamide	EMD Millipore	Cat.#S4117
Urea	Fisher Scientific	Cat.#BP169-212
Boric acid	Sigma-Aldrich	Cat.#B0394
Imidazole	Fisher Scientific	Cat.#O3190-500
HEPES	Sigma-Aldrich	Cat.#H4034
Potassium chloride	Sigma-Aldrich	Cat.#P9541
MES	Sigma-Aldrich	Cat.#M3671
Sodium hydroxide	Fisher Scientific	Cat.#S318-500
PEG4000	Sigma-Aldrich	Cat.#95904
EDTA	Sigma-Aldrich	Cat.#E5134
Sodium Cacodylate	Sigma-Aldrich	Cat.#20840
Blue dextran	Sigma-Aldrich	Cat.#D5751
Morpheus Halogen Mix (0.3 M NaF, 0.3 M NaBr, 0.3 M NaI)	Molecular Dimensions	Cat.#MD2-100-71
Mechlorethamine hydrochloride	Sigma-Aldrich	Cat.#122564
Carbenicillin	Fisher Scientific	Cat.#BP26481
**Software**		
GraphPad Prism 7.0c	https://www.graphpad.com	
ImageQuant	https://www.cytivalifesciences.com/en/us/products/items/imagequant-tl-analysis-software-p-28619?psmenu=2	
autoPROC	Vonrhein et al, 2011	
STARANISO	Vonrhein et al, 2018	
PHENIX	Adams et al, 2010	
Coot	Emsley et al, 2010	
Pymol	https://www.pymol.org/	
SBGrid	Morin et al, 2013	
**Other**		
1 kD MWCO Pre-wetted RC Tubing	Fisher Scientific	Cat.#08-670-12 C
HisPur Ni-NTA Resin	Thermo Scientific	Cat.#88222
Heparin Sepharose 6 Fast Flow Resin	Cytiva	Cat.#17099801
Superdex 200 Increase 10/300 GL	Cytiva	Cat.#10323523
MicroSpin G-25 Columns	Cytiva	Cat.#27532501
10 kD MWCO Amicon Ultra Centrifugal Filters	Merck Millipore	Cat.#UFC901024
30 kD MWCO Amicon Ultra Centrifugal Filters	Merck Millipore	Cat.#UFC503096
MRC 2 Well Crystallization Plate in UVXPO	Hampton Research	HR3-107
VDX Plate with sealant	Hampton Research	HR3-171
Siliconized circle cover slides	Hampton Research	HR3-231
96-well flat-bottom plate with Lid	CytoOne	CC7682-7596

### Vectors and cloning

Wild-type TfuAlkX (AAZ54292.1) and AbaAlkX (WP_000204287.1) genes were synthesized and cloned into a pBG102 vector (Vanderbilt University Center for Structural Biology) with codon optimization by GenScript. Mutants were generated by PCR using CloneAmp HiFi PCR premix (Takara) and primers containing the mutations. PCR products were digested with DpnI, gel purified with QIAquick Gel Extraction Kit (QIAGEN) and assembled using HiFi Assembly enzyme (NEB) prior to transformation into DH5α competent cells. All vectors were confirmed by Sanger or Oxford Nanopore sequencing.

For the bacterial growth assay, *AlkXA83P* and *Y300A* mutant allele expression constructs were generated by site-directed mutagenesis as follows. The pWH1266-*alkX* vector was amplified by PCR using Kapa HiFi HotStart polymerase (Roche) and primers encoding the indicated point mutants. The resulting PCR product was DpnI-digested and transformed into *E. coli* DH5α. The *alkXΔloop* mutant allele expression construct was generated by amplifying the *alkX* promoter and the *alkX* allele in which E298–R306 was replaced with GSSG from a gBlock (IDT). The resulting fragment was cloned into a SalI and BamHI-digested pWH1266 vector using HiFi Assembly (NEB). Plasmids harboring the desired mutations were screened by Sanger sequencing.

### Preparation of ICL DNA substrates

NM_5_-ICL and 2F-NM_8_-ICL DNA substrates used in ICL unhooking and DNA binding experiments were prepared as previously described (Bradley et al, 2020). For the NM_5_-ICL substrate, 26-bp oligodeoxynucleotides Cy5-d(TTTATTTTTATTTGACTTTTATTTTT) and FAM-d(AAAAATAAAAGTCAAATAAAAATAAA) were annealed at 200 μM in 40 mM sodium cacodylate (pH 7.0). The annealed duplex was incubated with three equivalents of mechlorethamine•HCl for 3 h at 37 °C in the dark. The reaction mix was desalted using MicroSpin G-25 columns (Cytiva) and gel-purified with 15% precast TBE-Urea gels (Thermo Fisher). Samples were mixed with loading buffer (5 mM EDTA (pH 8.0), 80% (wt/vol) formamide, 0.5 mg/ml Orange G) prior to loading. The gel was pre-run for 1 h at 200 V and run with samples for 1 h at 200 V. The NM_5_-ICL DNA band was excised and transferred to a 1-kDa MWCO dialysis tube (SpectrumLab), followed by electrophoresis at 100 V for 1 h. After dialysis in TE buffer overnight, the substrate was concentrated to 2 μM, aliquoted, and stored at −80 °C. 2F-NM_8_-ICL DNA substrates were prepared using the same procedure, with NM_8_ compound and oligodeoxynucleotides Cy5- TTTATTTTTATTTG^F^ACTTTTATTTTT and FAM-AAAAATAAAAG^F^TCAAATAAAAATAAA, where G^F^ is a ribo-fluoro-C2′ deoxyguanosine.

### Protein purification

AbaAlkX and TfuAlkX proteins were expressed in *E. coli* Tuner (DE3) cells grown in LB media containing 30 µg/mL kanamycin. Expression was induced with 0.1 mM IPTG (isopropyl-β-D-thiogalactopyranoside) at an OD_600_ of 0.8. Selenomethionine-derivatized (SeMet) TfuAlkX was overexpressed in *E. coli* Tuner (DE3) cells with 0.1 mM IPTG induction in M9 media containing 0.1 g/L lysine, 0.1 g/L phenylalanine, 0.1 g/L threonine, 0.05 g/L leucine, 0.05 g/L isoleucine, 0.05 g/L valine, and 0.05 g/L selenomethionine. After growing for 16 h at 16 °C, cells were harvested, homogenized in lysis buffer (50 mM Tris (pH 8.0), 500 mM NaCl, 1 mM Tris (2-carboxyethyl) phosphine (TCEP), 1 mM phenylmethylsulfonyl fluoride (PMSF), 25 mM imidazole, 10% glycerol) and lysed by sonication. Cell debris was removed by centrifugation at 21,000 rpm for 30 min at 4 °C. The supernatant was loaded onto an Ni-NTA affinity column, and the His_6_-tagged protein was eluted with buffer B (50 mM Tris (pH 8.0), 500 mM NaCl, 250 mM imidazole, 10% glycerol). Protein fractions were pooled and supplemented with 0.1 mM EDTA and 1 mM TCEP before incubation with 1 mg rhinovirus 3C protease (GST-tagged) at 4 °C overnight. The cleaved protein was diluted fivefold with buffer C (50 mM Tris (pH 8.0), 10% glycerol, 0.1 mM EDTA, 1 mM TCEP) and purified on a heparin sepharose column (Cytiva) with a 0–1 M NaCl/buffer C linear gradient. Fractions were pooled and repassed over the Ni-NTA affinity column. The flow-through was concentrated using a 10-kDa MWCO Amicon Ultra filter (Millipore). The concentrated protein was then passed over a Superdex 200 column (Cytiva) in 25 mM Tris (pH 8.0), 150 mM NaCl, 5% glycerol, 0.1 mM EDTA, and 1 mM TCEP. AbaAlkX and TfuAlkX were concentrated to 5 mg/mL and 0.6 mg/mL, respectively, flash-frozen in liquid nitrogen, and stored at −80 °C.

### Crystallization, X-ray data collection, and structure determination

THF-DNA was prepared by annealing d(TGAGTCGT(THF)GATGACCAC) and d(GTGGTCATCCACGACTCA) at 500 μM in the presence of two equivalents of N7-methylguanine in 10 mM MES (pH 6.5) and 40 mM NaCl. For crystallization, SeMet-TfuAlkX was incubated with 1.2 equivalents of THF-DNA on ice for 20 min and concentrated to 1.5 mg/ml (protein concentration) using a 30-kDa MWCO Amicon Ultra Centrifugal Filter. Block-shaped crystals were observed in hanging drop vapor diffusion plates after 3 days in 0.1 M MES/imidazole (pH 6.5), 10% PEG 4000, 10% glycerol, 0.03 M sodium fluoride, 0.03 M sodium bromide, and 0.03 M sodium iodide. Crystals were transferred to a cryoprotectant solution consisting of mother liquor supplemented with 15–20% (v/v) glycerol and flash-frozen in liquid nitrogen for data collection.

X-ray diffraction data (Table EV1) were collected at the European Synchrotron Radiation Facility (ESRF) beamline ID30A (λ = 0.96546 Å). The datasets were processed using autoPROC (Vonrhein et al, 2011) and STARANISO (Vonrhein et al, 2018). Phases were determined by single-wavelength anomalous dispersion (SAD). Thirteen selenium sites were identified in the asymmetric unit by HySS in the PHENIX package (Adams et al, 2010) and used for model building through AutoSol and Autobuild. Iterative cycles of model rebuilding and refinement were carried out using COOT (Emsley et al, 2010) and PHENIX (Adams et al, 2010). MolProbity was used to assess the overall quality of the structural models (Davis et al, 2007). Structure figures were made using PyMol 3.0 (Schrödinger, LLC). The RMSD was calculated using TM align in the RCSB online tool (Bittrich et al, 2024). All structural biology software was curated by SBGrid (Morin et al, 2013).

### Base excision assay

Glycosylase reactions were carried out at room temperature with 1 µM enzyme and 50 nM DNA substrate in glycosylase buffer (50 mM HEPES, pH 7.5, 100 mM KCl, 10 mM EDTA, and 10% glycerol). At various time points, 4 µL aliquots were taken and added to 1 µL of 1 M NaOH. The samples were heated at 55 °C for 2 min, followed by the addition of 5 µL of denaturing loading buffer (5 mM EDTA (pH 8.0), 80% formamide, 1 mg/ml blue dextran) and heating at 55 °C for 5 min to avoid spontaneous depurination at high temperature. All samples were electrophoresed on a 20% acrylamide/8 M urea denaturing gel at 40 W for 2 h in 0.5× TBE buffer. Gels were imaged on an Amersham Typhoon biomolecular imagers RGB (Cytiva) using the Cy5 (655–685 nm) channel. Bands were quantified with ImageQuant (Cytiva). ICL unhooking (% product) was calculated as (MA + β/δ-elim) / (ICL + MA + β/δ-elim), where ICL, MA, and β/δ-elim are the amounts of ICL substrate, monoadduct product, and AP β/δ-elimination products, respectively.

### Electrophoretic mobility shift assay (EMSA)

DNA binding reactions were carried out by incubating 4 µL protein at varying concentrations with 4 µL 20 nM 2F-NM_8_-ICL DNA substrate at room temperature for 20 min in binding buffer (25 mM Tris (pH 7.5), 100 mM NaCl, 5% glycerol, 0.1 mM EDTA, 1 mM TCEP). Each sample was mixed with 4 µL of 75% glycerol and then loaded onto a 5% native TBE gel. The gel was pre-run at 200 V for 1 h and run with samples at 200 V for 1 h. Gels were imaged on an Amersham Typhoon biomolecular imagers RGB (Cytiva) using the Cy5 channel and bands quantified with ImageQuant (Cytiva).

### Bacterial growth curves

Overnight cultures of WT and ∆*alkX A. baumannii* strains harboring the indicated plasmids were started from single colonies in LB broth, with 75 µg/mL carbenicillin. The following day, overnight cultures were back-diluted into fresh LB media with carbenicillin 1:50, and allowed to grow with shaking at 37 °C for 1 h. Back-diluted cultures were used to inoculate the wells of 96-well microtiter plates 1:100 with carbenicillin ±33 µM mechlorethamine-HCL. Plates were cultured with continuous shaking at 37 °C for 18 h and OD_600_ was recorded every 60 min.

## Supplementary information

Table EV1

Appendix

Peer Review File

Source data Fig. 2

Source data Fig. 3

Source data Fig. 4

Expanded View Figures

## Data Availability

Structure factors and corresponding atomic coordinates for the TfuAlkX/DNA crystal structure have been deposited in the PDB under accession code 9ZD6. The source data of this paper are collected in the following database record: biostudies:S-SCDT-10_1038-S44319-026-00785-6.
