# Supplementary material for: DNA binding and lesion recognition by the bacterial interstrand DNA crosslink glycosylase AlkX
Source: EMBO Rep. 2026 May 8;27(12):3173–88. doi: 10.1038/s44319-026-00785-6 (PMC13303869; doi:10.1038/s44319-026-00785-6)
Supplement: Supplementary file 1 — Table EV1 [file 44319_2026_785_MOESM1_ESM.docx]

| **Table EV1. X-ray data collection and refinement statistics** | |
| --- | --- |
| **Data collection** |  |
| Space group | P2_1_2_1_2_1_ |
| Unit cell |  |
| *a, b, c* (Å) | 56.60, 115.64, 160.68 |
| *⍺, β, γ* (°) | 90, 90, 90 |
| Wavelength (Å) | 0.9655 |
| Resolution range (Å) | 56.60 - 2.60 (2.72 - 2.60)^a^ |
| Total reflections | 214983 (25797) |
| Unique reflections | 33291 (4018) |
| *R*_merge_ | 0.135 (0.983) |
| *R*_meas_ | 0.147 (1.068) |
| *R*_pim_ | 0.058 (0.413) |
| Mean I/σ(I) | 8.8 (2.3) |
| Completeness (%) | 99.9 (99.8) |
| Redundancy | 6.5 (6.4) |
| Wilson B-factor (Å^2^) | 50.83 |
| CC_1/2_ | 0.994 (0.801) |
| **Refinement** |  |
| Resolution (Å) | 50.84 - 2.60 (2.69 - 2.60) |
| No. reflections | 33,197 (3,287) |
| *R*_work_ | 0.2068 (0.3149) |
| *R*_free_^b^ | 0.2555 (0.3527) |
| No. atoms | 6,892 |
| Protein | 6,111 |
| DNA | 721 |
| Water | 55 |
| Other | 5 |
| Avg. B-factor (Å^2^) |  |
| Protein | 64.49 |
| DNA | 71.74 |
| Water | 55.69 |
| Other | 69.86 |
| Ramachandran distribution |  |
| Favored (%) | 96.2 |
| Allowed (%) | 3.7 |
| Outliers (%) | 0.1 |
| RMS bonds (Å) | 0.008 |
| RMS angles (°) | 1.05 |
| ^a^ Statistics for the highest resolution shell are shown in parentheses  ^b^ *R*_free_ was determined from the 5% of reflections excluded from refinement | |
