# Supplementary material for: DNA binding and lesion recognition by the bacterial interstrand DNA crosslink glycosylase AlkX
Source: EMBO Rep. 2026 May 8;27(12):3173–88. doi: 10.1038/s44319-026-00785-6 (PMC13303869; doi:10.1038/s44319-026-00785-6)
Supplement: Supplementary file 2 — Appendix [file 44319_2026_785_MOESM2_ESM.pdf]

Appendix

DNA binding and lesion recognition by the bacterial interstrand DNA crosslink glycosylase AlkX

Yujuan Cai (蔡毓娟)†, Dillon E. Kunkle†, Marcel D. Edinbugh, Noah P. Bradley, Eric P. Skaar*, and Brandt F. Eichman*

Table of Content

	page
Appendix Figure S1	1
Appendix Figure S2	2
Appendix Figure S3	3

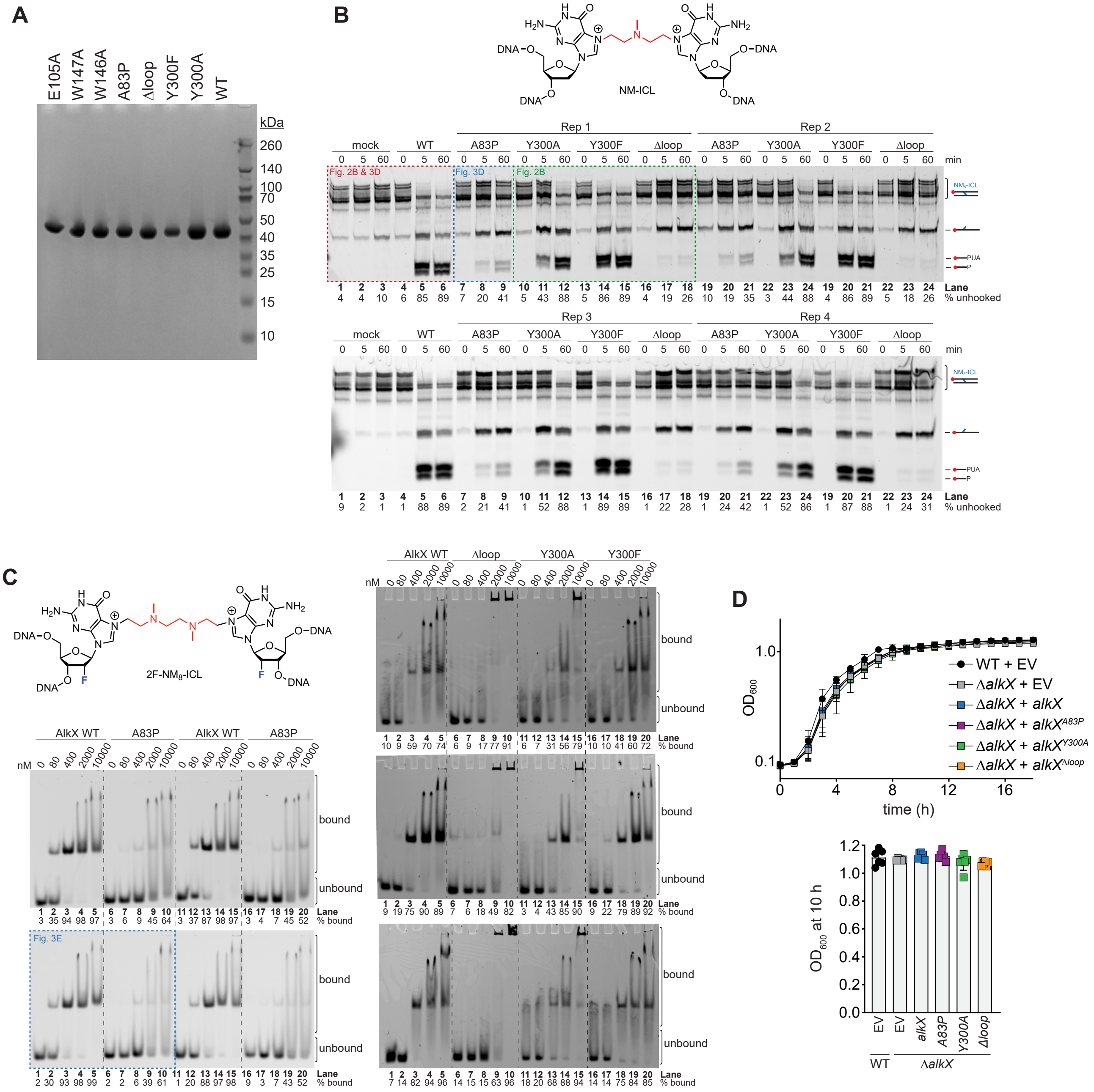

Appendix Figure S1. Biochemical characterization of AbaAlkX mutants. **A**. SDS-PAGE of purified AbaAlkX proteins. **B**. The chemical structure of the NM_5-ICL used in base excision activity assays is shown at the top. Fluorescence (Cy5) scans of denaturing PAGE gels with four replicates of AbaAlkX reactions containing Cy5-labeled NM_5-ICL DNA at 0, 5, and 60 min. Schematics to the right of the gel define the regions used in quantification. The AP-site product is cleaved during NaOH work-up to form a 3′-phospho-α,β-unsaturated aldehyde (PUA) and a 3′-phosphate (P). Sections of the gels shown in Figures 2B and 3D are indicated by colored dashed boxes. **C**. The chemical structure of 2F-NM_8-ICL DNA used in electrophoretic mobility shift assays (EMSAs) and fluorescence (Cy5) scans of native PAGE gels containing 3-4 replicates of AbaAlkX binding to Cy5-labeled 2F-NM_8-ICL DNA. The blue dashed box indicates the section of the gel shown in Figure 3E. **D**. AlkX complementation studies in untreated conditions. *Top*: Growth curves of *A. baumannii* strains (WT and *ΔalkX*) harboring pWH1266 empty vector (EV) or indicated pWH1266-*alkX* expression vectors in LB media. The OD_{600} was recorded every 60 min. Data represent the mean ± SD of at least 6 biological replicates performed in technical triplicate. *Bottom*: OD_{600} values at 10 hr growth. Data represent the mean ± SD. Each dot represents an individual biological replicate, performed in technical triplicate.

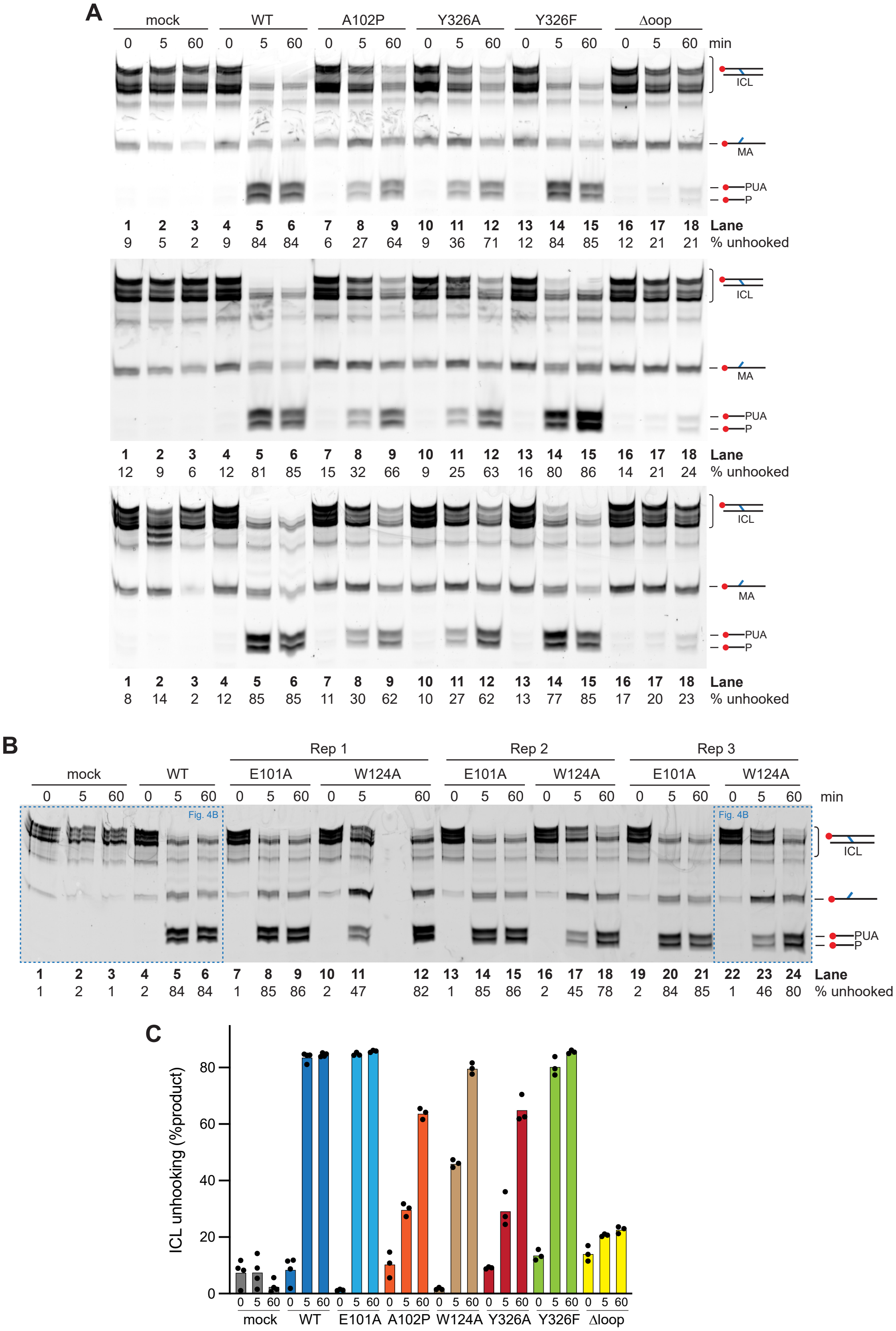

Appendix Figure S2. ICL unhooking activity of TfuAlkX mutants. **A,B**. Denaturing PAGE gels (Cy5 fluorescence scans) of reactions between TfuAlkX mutants and Cy5-labeled NM-ICL DNA, showing three biological replicates of A102P, Y326A, Y326F, Δloop (A) and E101A and W124A (B). The blue dashed boxes indicate the sections of the gel shown in Figure 4B. **C**. Quantification of the gels.

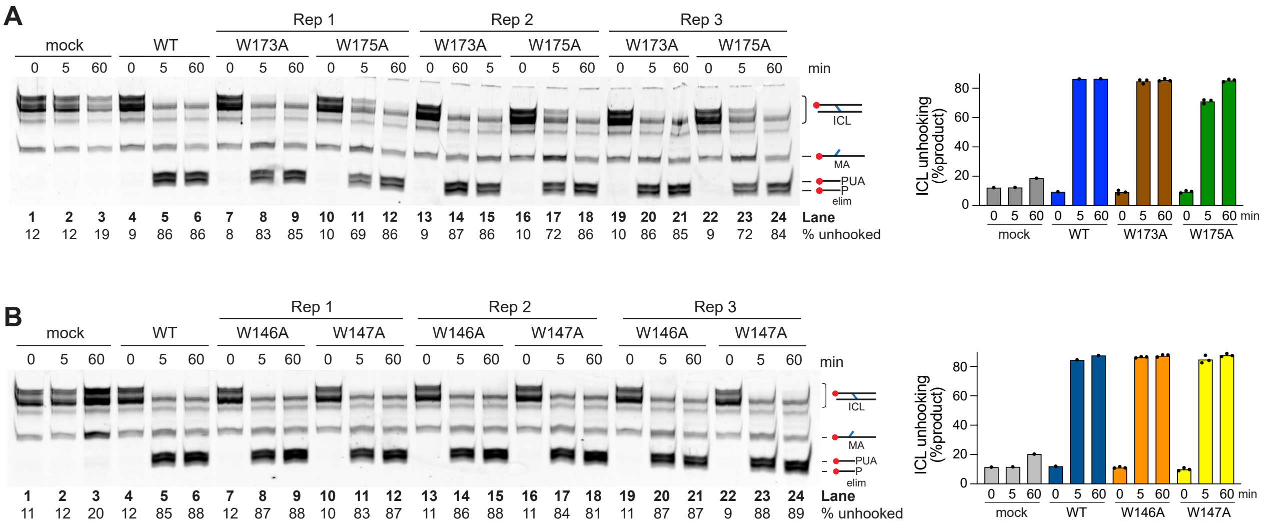

Appendix Figure S3. ICL unhooking activity of residues in the αI region. Denaturing PAGE gels of three biological replicates of NM-ICL unhooking reactions containing TfuAlkX (A) and AbaAlkX (B) mutants. Quantification of the gels is shown in the plots on the right.
