# Supplementary figures and images for: DNA binding and lesion recognition by the bacterial interstrand DNA crosslink glycosylase AlkX
Source: EMBO Rep. 2026 May 8;27(12):3173–88. doi: 10.1038/s44319-026-00785-6 (PMC13303869; doi:10.1038/s44319-026-00785-6)

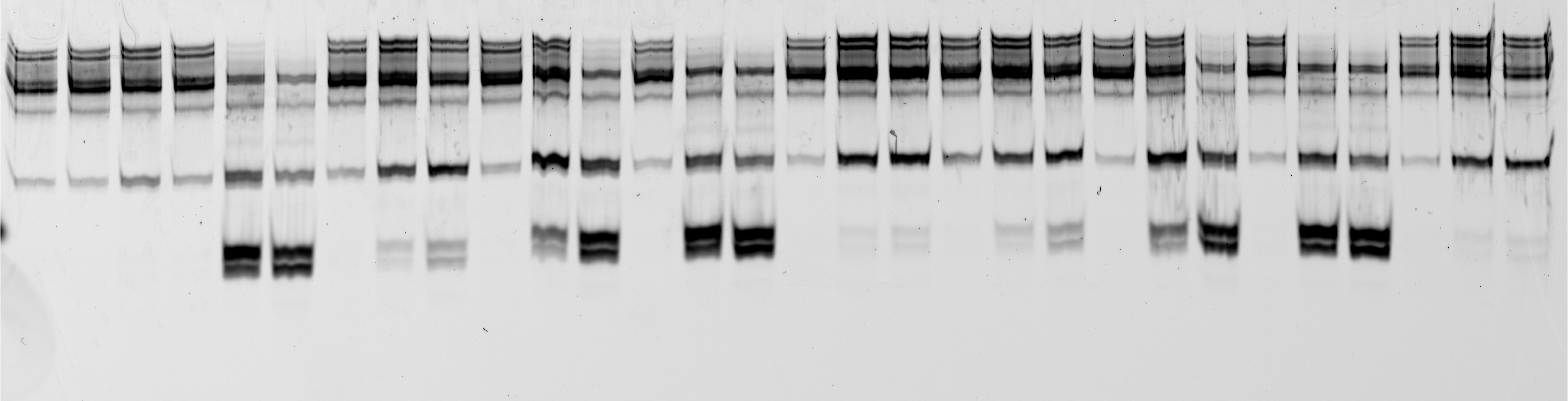

Supplement: Supplementary file 4 — Source data Fig. 2 [file 44319_2026_785_MOESM4_ESM.zip › Figure 2/2B/DenaturingGelReplicate1-2.png]

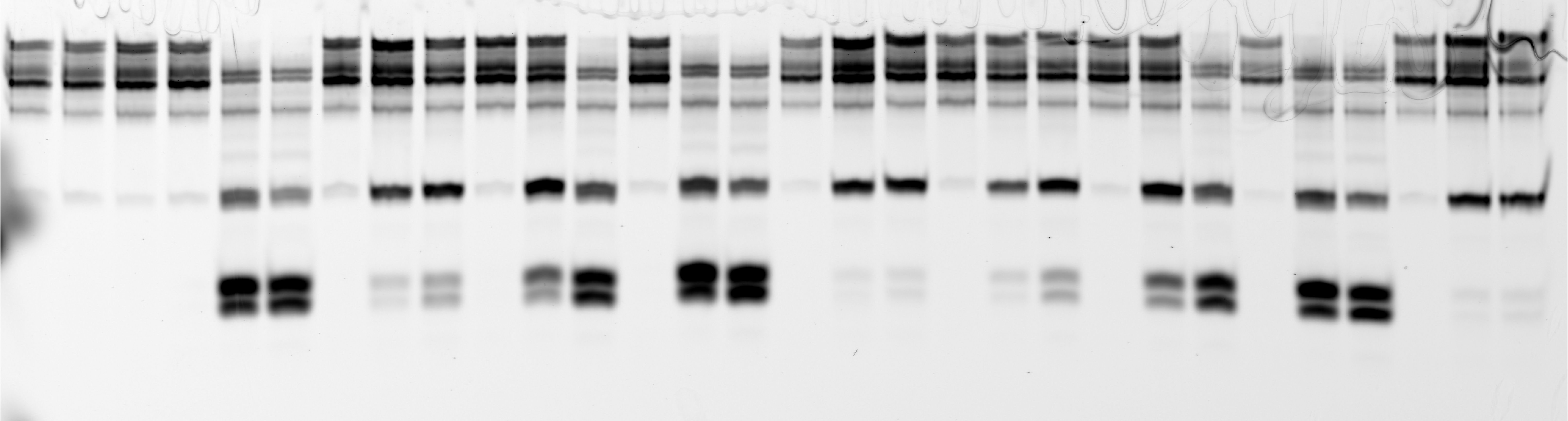

Supplement: Supplementary file 4 — Source data Fig. 2 [file 44319_2026_785_MOESM4_ESM.zip › Figure 2/2B/DenaturingGelReplicate3-4.png]

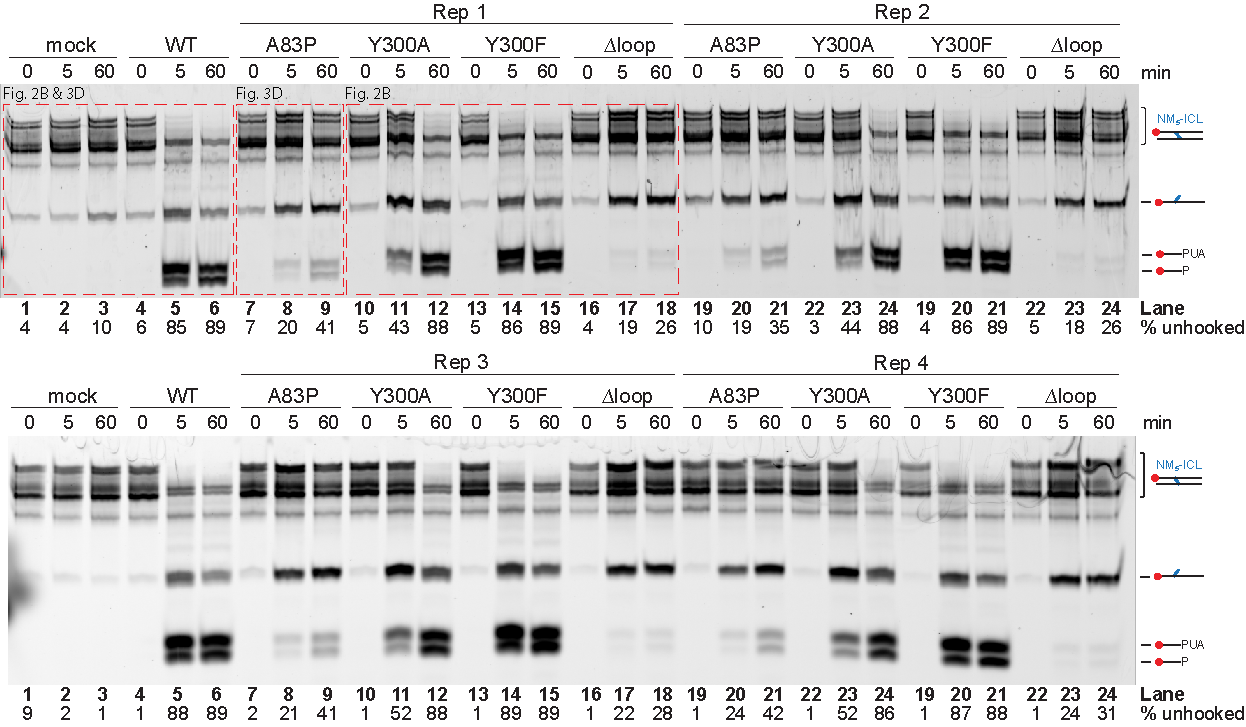

Supplement: Supplementary file 4 — Source data Fig. 2 [file 44319_2026_785_MOESM4_ESM.zip › Figure 2/2B/ReadMe.docx]

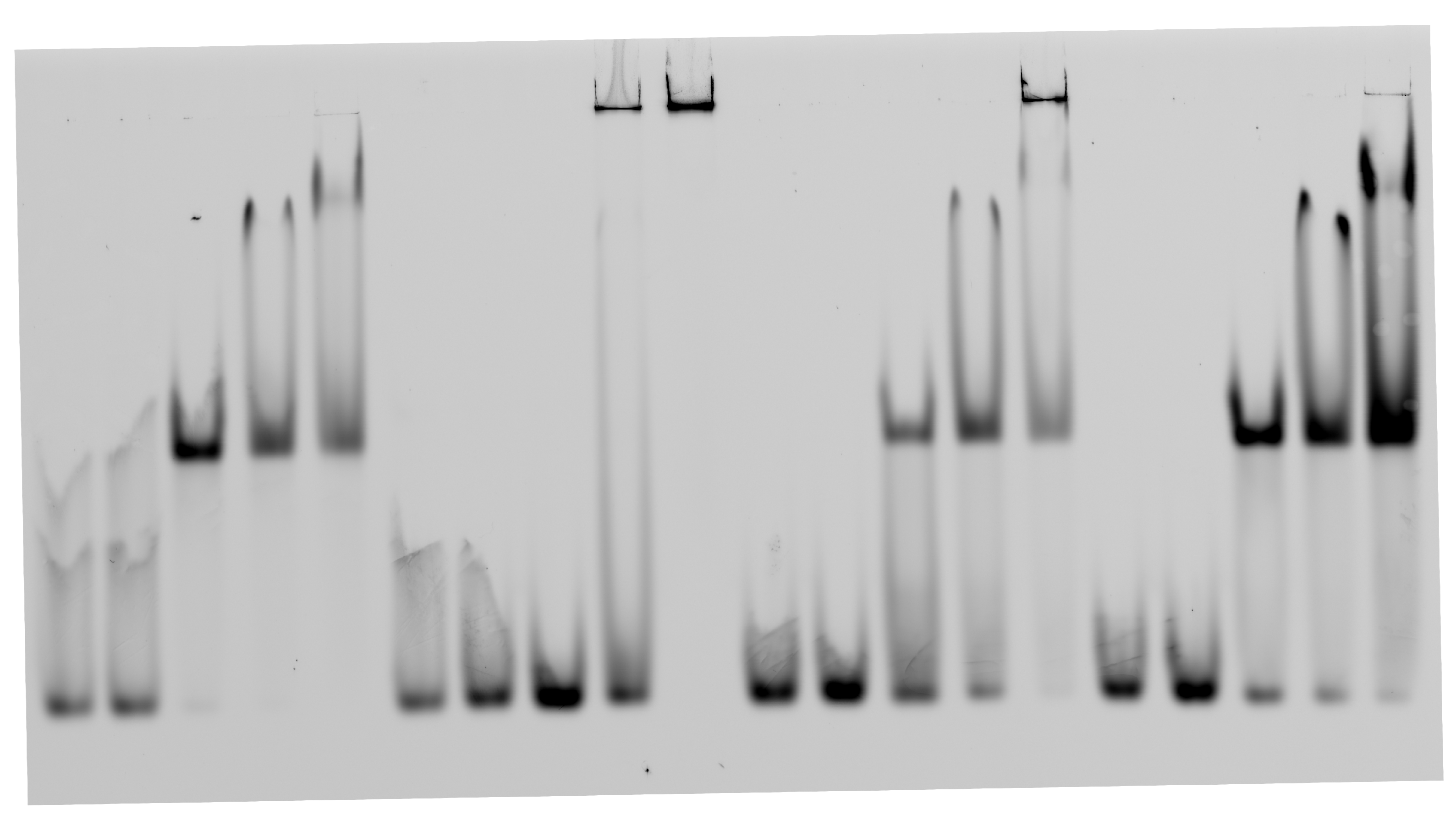

Supplement: Supplementary file 4 — Source data Fig. 2 [file 44319_2026_785_MOESM4_ESM.zip › Figure 2/2C/EMSAGelReplicate1.png]

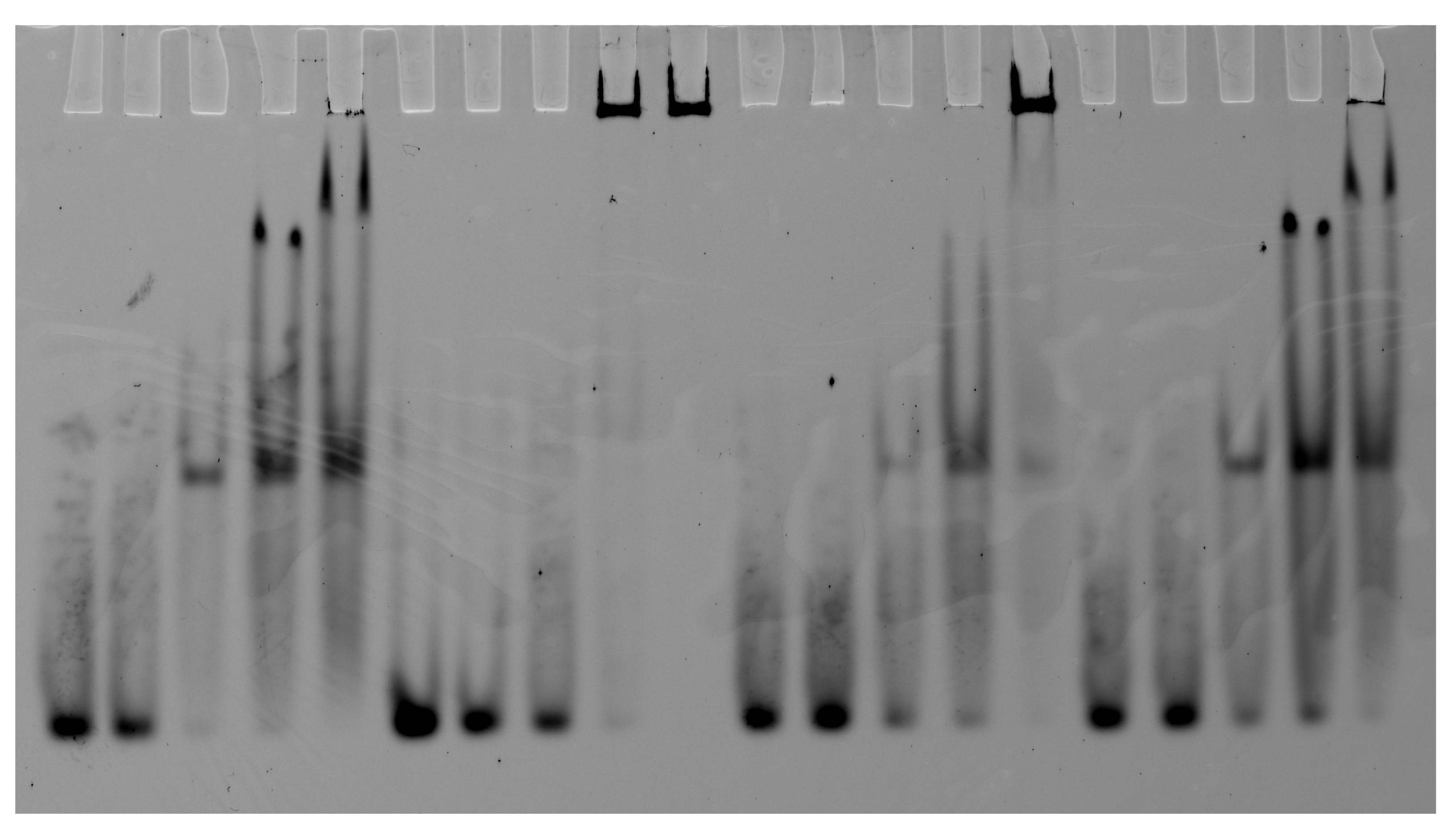

Supplement: Supplementary file 4 — Source data Fig. 2 [file 44319_2026_785_MOESM4_ESM.zip › Figure 2/2C/EMSAGelReplicate2.png]

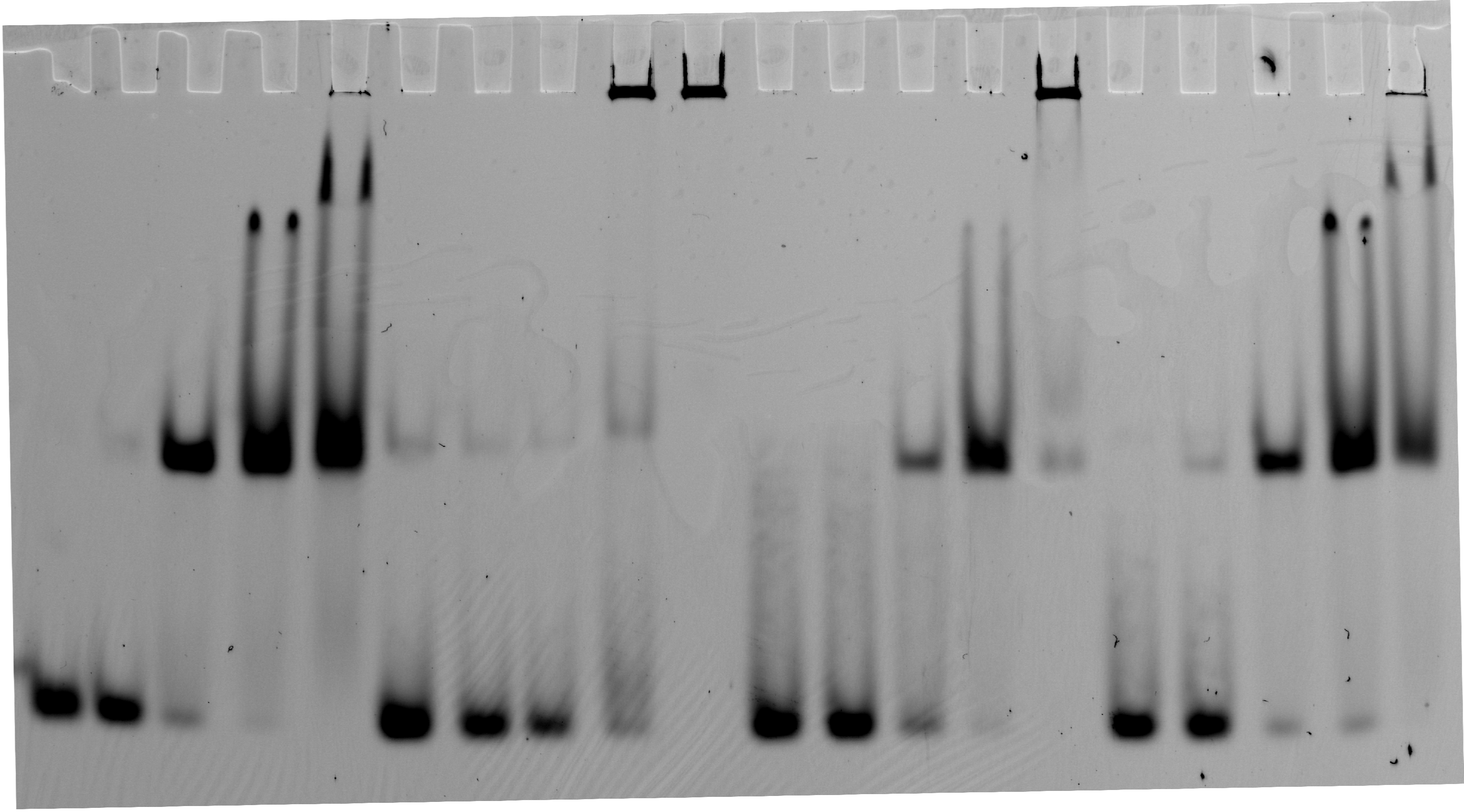

Supplement: Supplementary file 4 — Source data Fig. 2 [file 44319_2026_785_MOESM4_ESM.zip › Figure 2/2C/EMSAGelReplicate3.png]

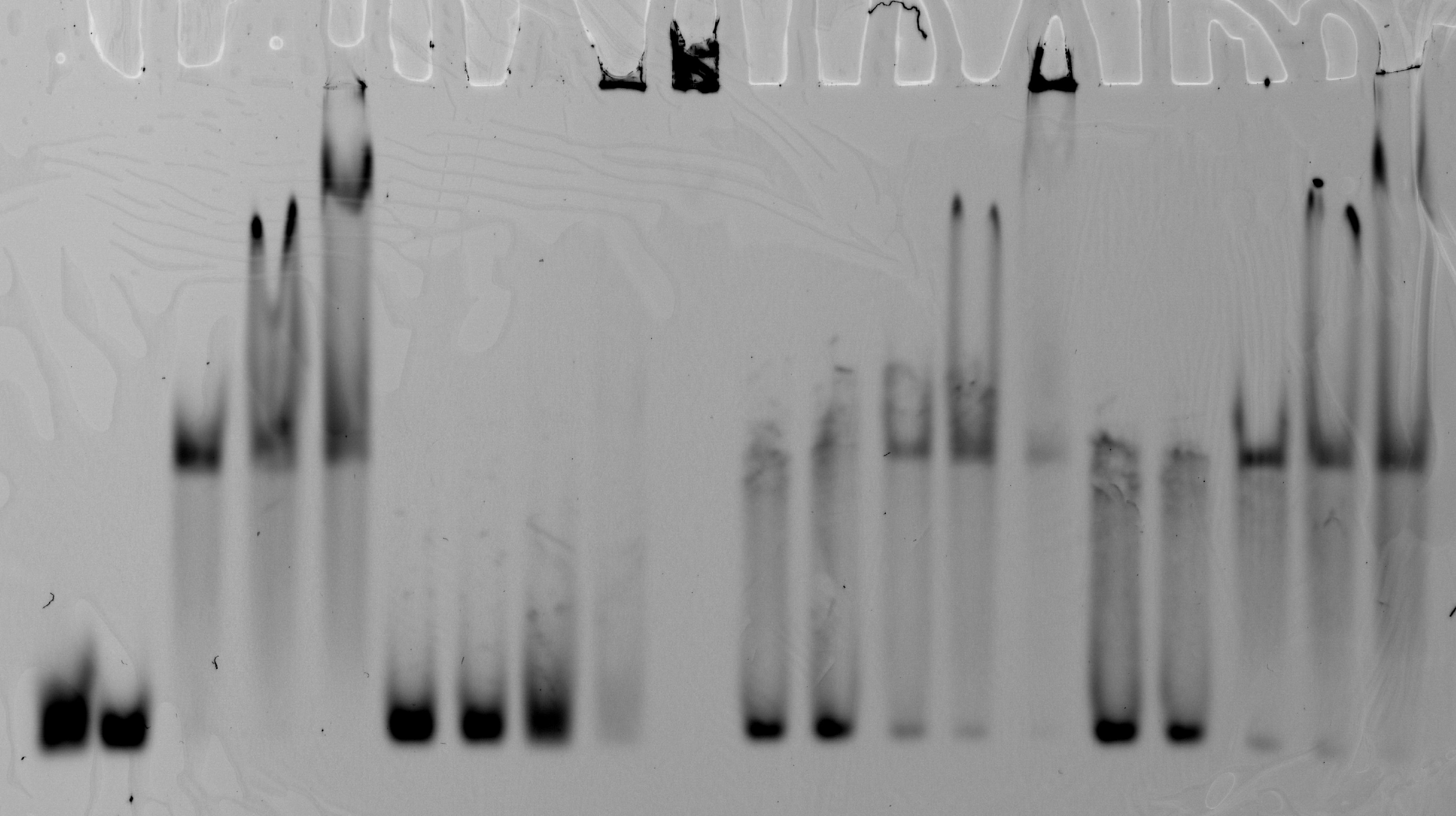

Supplement: Supplementary file 4 — Source data Fig. 2 [file 44319_2026_785_MOESM4_ESM.zip › Figure 2/2C/EMSAGelReplicate4.png]

EMSAGelReplicate-1 was shown in Figure 2C.


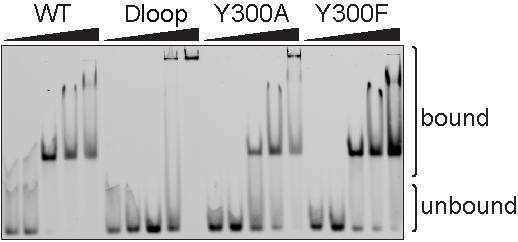


Other replicates was shown in EV figures.


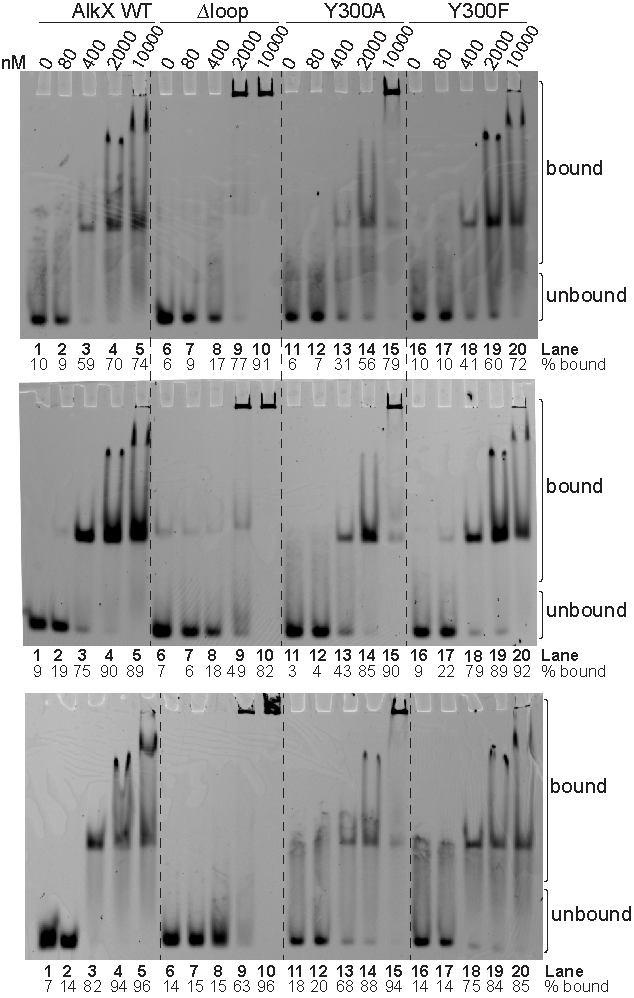

Supplement: Supplementary file 4 — Source data Fig. 2 [file 44319_2026_785_MOESM4_ESM.zip › Figure 2/2C/ReadMe.docx]

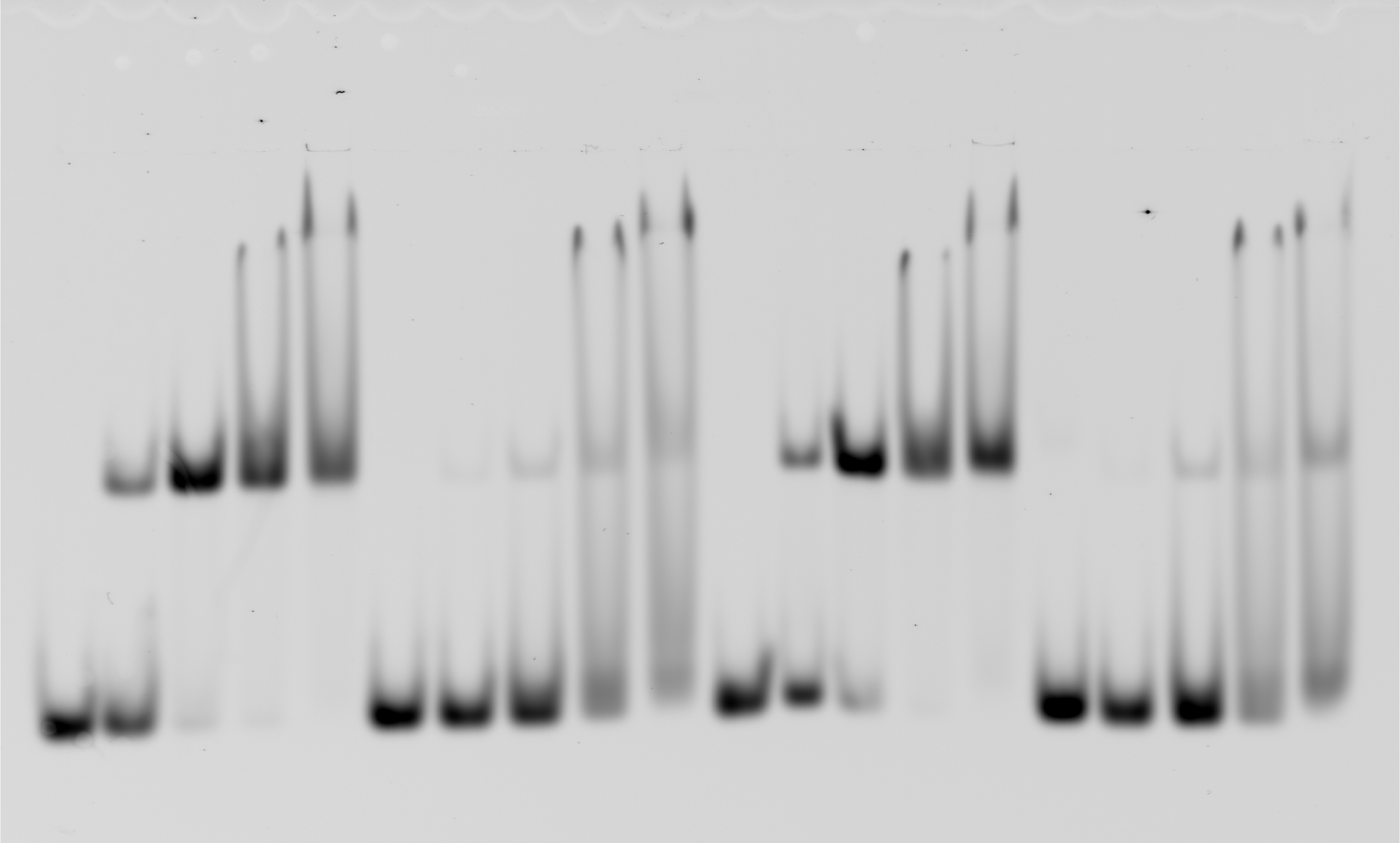

Supplement: Supplementary file 5 — Source data Fig. 3 [file 44319_2026_785_MOESM5_ESM.zip › Figure 3/3E/EMSAReplicate1-2.png]

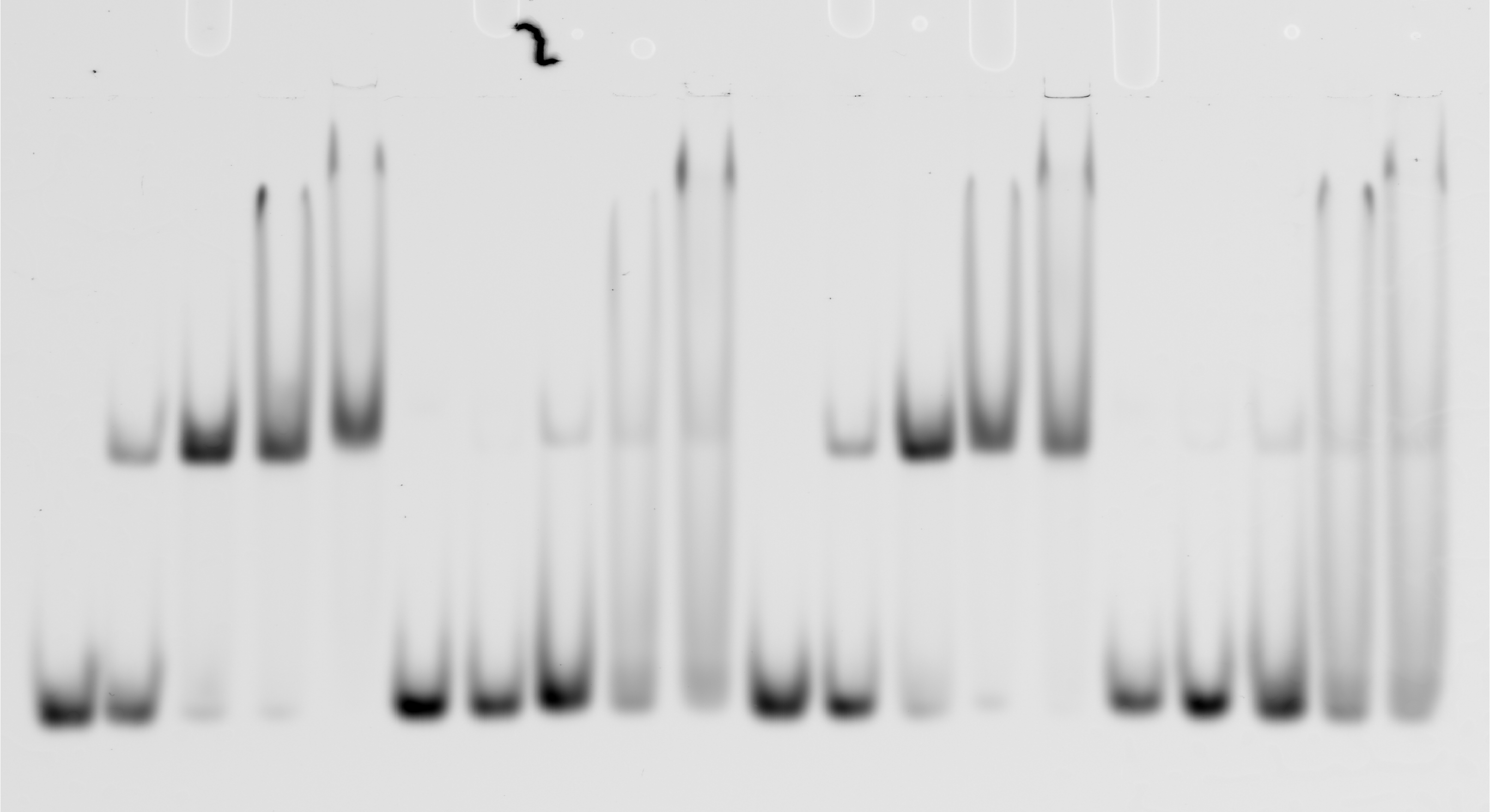

Supplement: Supplementary file 5 — Source data Fig. 3 [file 44319_2026_785_MOESM5_ESM.zip › Figure 3/3E/EMSAReplicate3-4.png]

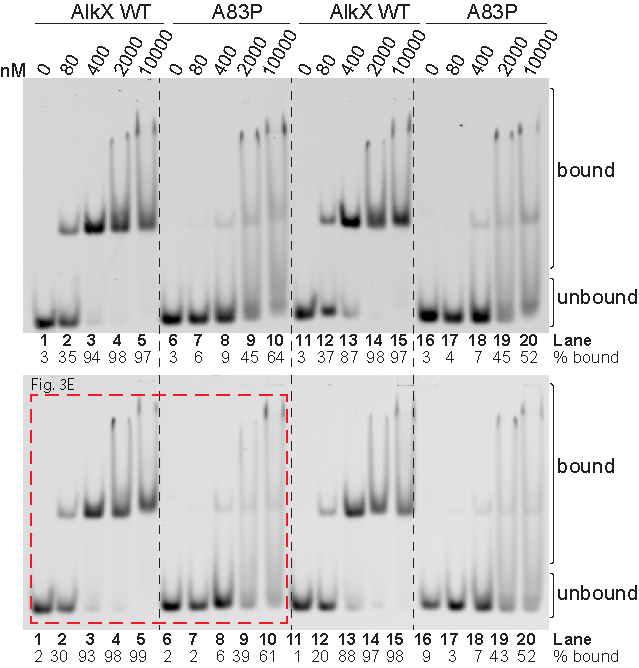

Supplement: Supplementary file 5 — Source data Fig. 3 [file 44319_2026_785_MOESM5_ESM.zip › Figure 3/3E/ReadMe.docx]

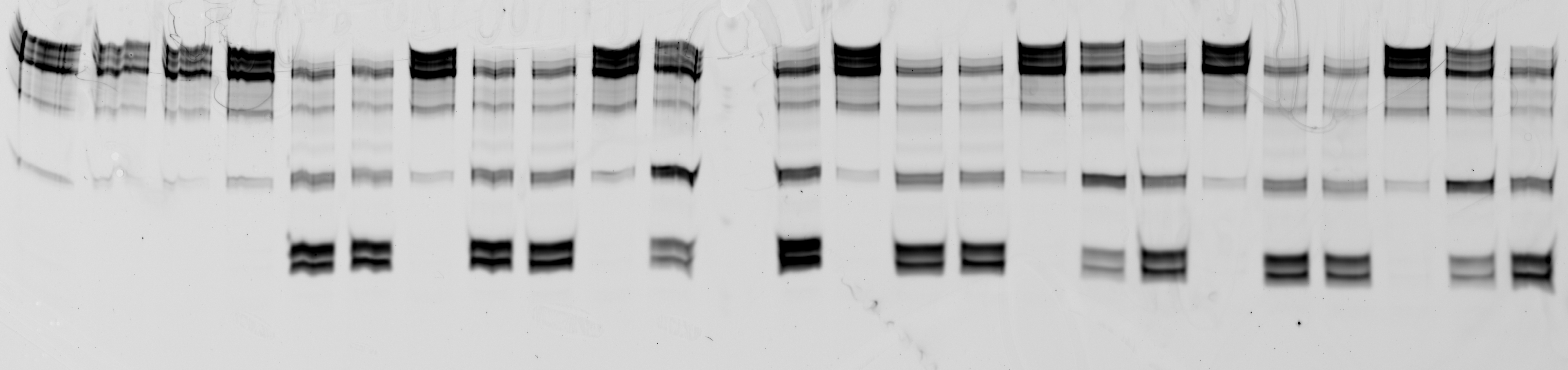

Supplement: Supplementary file 6 — Source data Fig. 4 [file 44319_2026_785_MOESM6_ESM.zip › Figure 4/4B/DenaturingGelsReplicate1-3.png]

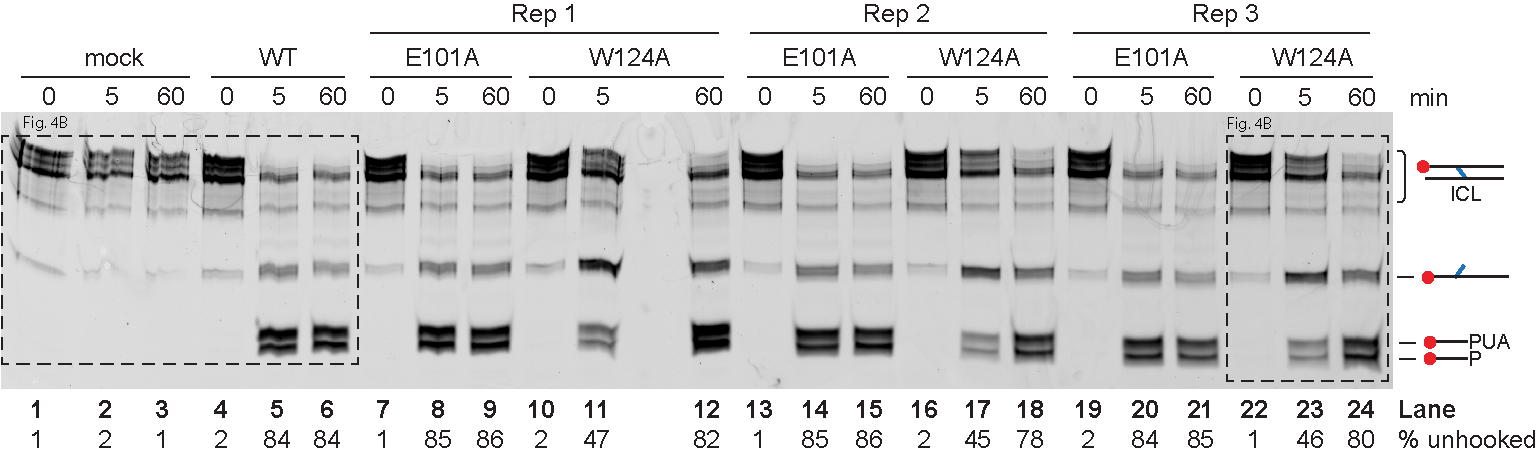

Supplement: Supplementary file 6 — Source data Fig. 4 [file 44319_2026_785_MOESM6_ESM.zip › Figure 4/4B/ReadMe.docx]
