# Supplementary material for: DNA binding and lesion recognition by the bacterial interstrand DNA crosslink glycosylase AlkX
Source: EMBO Rep. 2026 May 8;27(12):3173–88. doi: 10.1038/s44319-026-00785-6 (PMC13303869; doi:10.1038/s44319-026-00785-6)
Supplement: Supplementary file 7 — Expanded View Figures [file 44319_2026_785_MOESM7_ESM.pdf]

Expanded View Figures

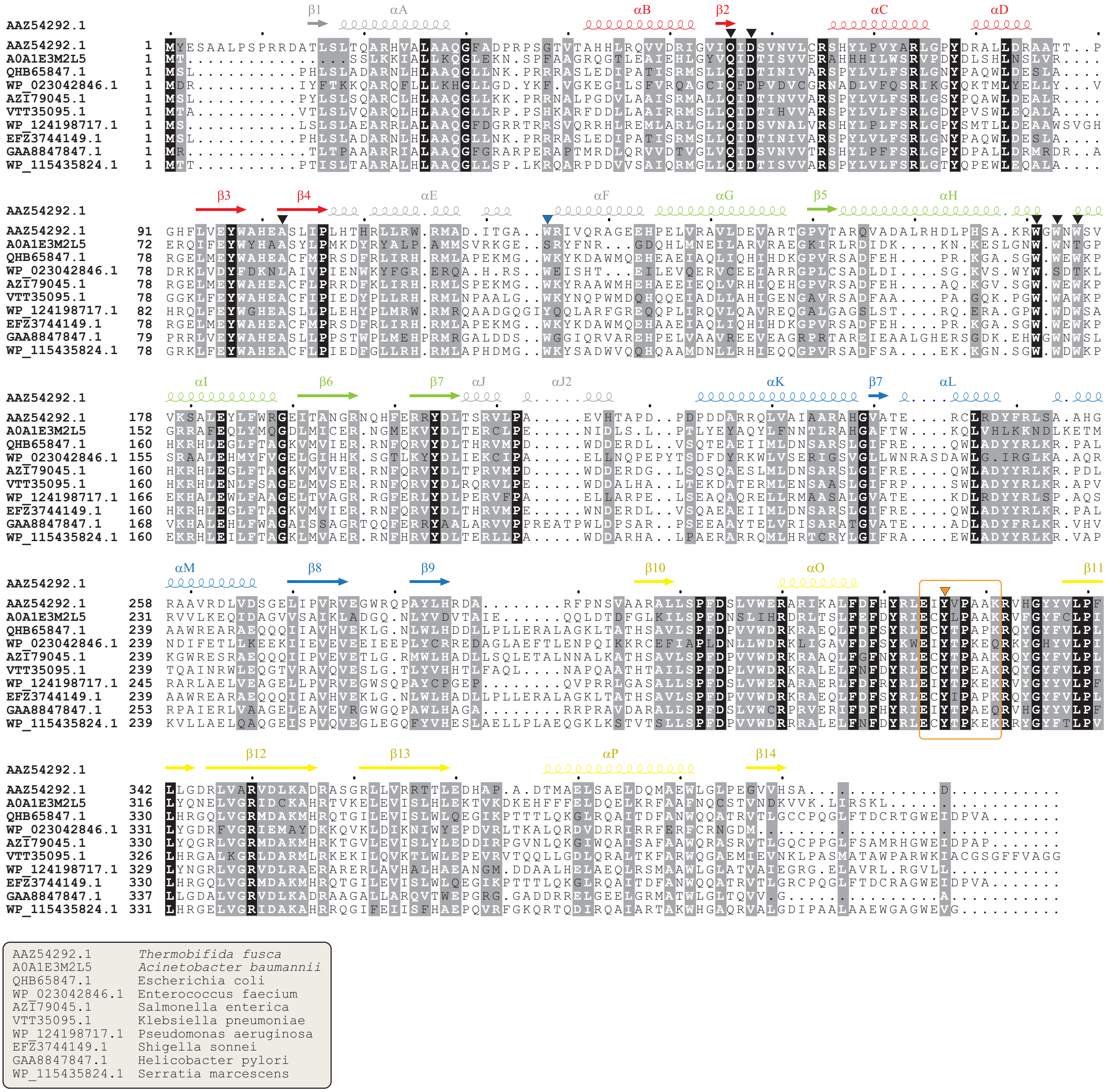

Figure EV1. Sequence alignment of YQL/AlkX proteins.

Secondary structure elements are colored by domain as in Fig. 1C. The DBL is highlighted with an orange box. Triangles denote residues mutated in this study. The orange and blue triangles designate the DBL tyrosine and the putative ICL recognition motif, respectively. The organisms corresponding to the sequence accession numbers are shown at the bottom of the figure.

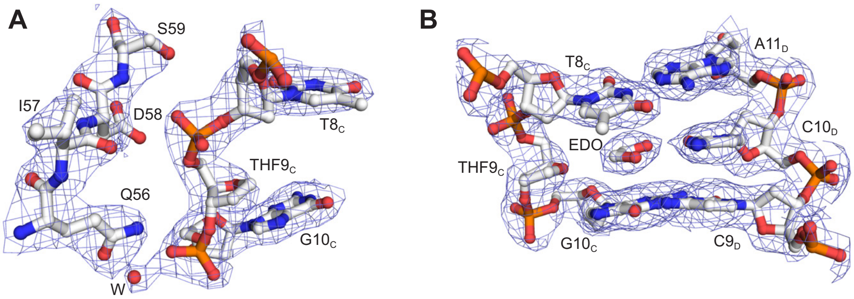

Figure EV2. Electron density.

Interaction of the THF region with the active site (**A**) and the opposite DNA strand (**B**). 2Fo-Fc electron density contoured to 1σ is superimposed.

 © The Author(s)

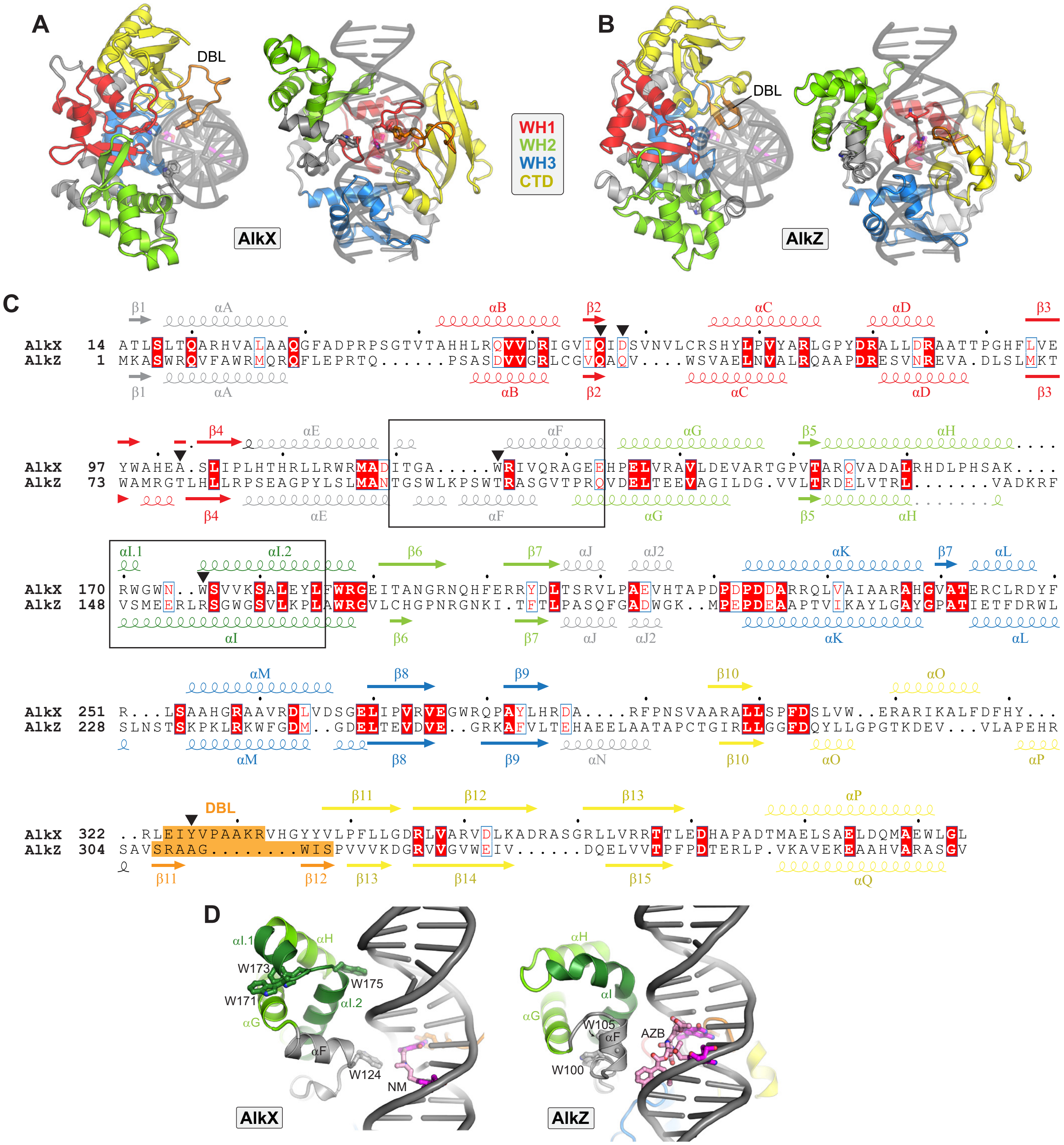

Figure EV3. Comparison of AlkX and AlkZ structures.

(**A**) Crystal structure of TfuAlkX bound to THF-DNA. The protein is colored by domain (WH1 red, WH2 green, WH3 blue, C-terminal domain yellow, DBL orange). (**B**) Crystal structure of *Streptomyces sahachiroi* (Ssa) AlkZ (PDB ID 5UUJ) docked against THF-DNA from the TfuAlkX structure. (**C**) Sequence alignment of TfuAlkX and SsaAlkZ. Putative lesion-sensing regions αF and αI are boxed. Residues important for ICL unhooking in AlkX are marked with black triangles. (**D**) Comparison of αF and WH2 regions of AlkX (left) and AlkZ (right). DNA models of NM- and azinomycin B (AZB)-ICLs are based on the DNA in the TfuAlkX structure.

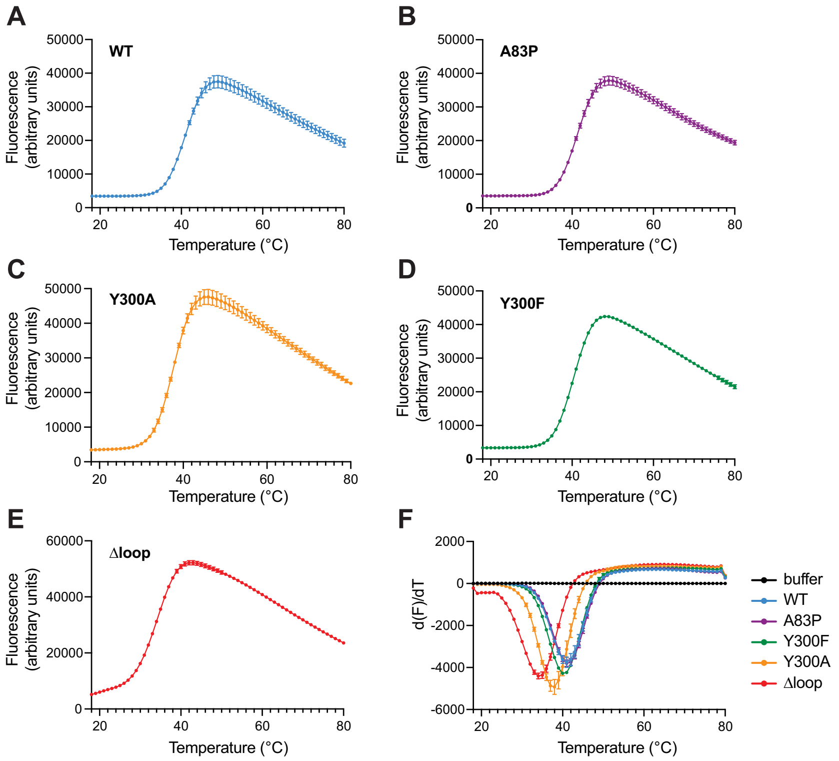

Figure EV4. Thermostability of AlkX mutants.

(**A–E**) Differential scanning fluorescence thermal denaturation profiles for AlkX WT (**A**), A83P (**B**), Y300A (**C**), Y300F (**D**), and Δloop (**E**). Data represent the mean ± SD ($n = 3$). Samples contained 10 μM protein, 20 mM Tris, pH 8.0, 150 mM NaCl, 1 mM TCEP, 0.1 mM EDTA, and 0.5× SYPRO Orange and measurements were carried out as previously described (Dorival et al, 2025). (**F**) First derivatives of the thermal denaturation data in panels (**A–E**). The minima signify the melting temperatures (T_m). Data represent the mean ± SD ($n = 3$).

© The Author(s)
