# Supplementary material for: DNA binding and lesion recognition by the bacterial interstrand DNA crosslink glycosylase AlkX
Source: EMBO Rep. 2026 May 8;27(12):3173–88. doi: 10.1038/s44319-026-00785-6 (PMC13303869; doi:10.1038/s44319-026-00785-6)
Supplement: Supplementary file 3 — Peer Review File [file 44319_2026_785_MOESM3_ESM.pdf]

EMBO reports

DNA binding and lesion recognition by the bacterial interstrand DNA crosslink glycosylase AlkX

Yujuan Cai, Dillon Kunkle, Marcel Edinbugh, Noah Bradley, Eric Skaar, and Brandt Eichman

Corresponding author(s): Brandt Eichman (brandt.eichman@vanderbilt.edu) , Eric Skaar (eric.skaar@vumc.org)

Review Timeline:

Submission Date:	8th Dec 25
Editorial Decision:	23rd Feb 26
Revision Received:	2nd Mar 26
Editorial Decision:	23rd Mar 26
Revision Received:	26th Mar 26
Accepted:	13th Apr 26

Editor: Esther Schnapp

Transaction Report:

(Note: With the exception of the correction of typographical or spelling errors that could be a source of ambiguity, letters and reports are not edited. Depending on transfer agreements, referee reports obtained elsewhere may or may not be included in this compilation. Referee reports are anonymous unless the Referee chooses to sign their reports.)

Dear Dr. Eichman,

Thank you for the submission of your manuscript to EMBO reports. We have now received the full set of referee reports that is pasted below.

As you will see, all referees acknowledge that the findings are interesting and only have minor comments that should all be addressed for the publication of your study here.

I would thus like to invite you to revise your manuscript with the understanding that the referee concerns must be fully addressed and their suggestions taken on board. Please address all referee concerns in a complete point-by-point response. Acceptance of the manuscript will depend on a positive outcome of a second round of review. It is EMBO reports policy to allow a single round of major revision only and acceptance or rejection of the manuscript will therefore depend on the completeness of your responses included in the next, final version of the manuscript.

We realize that it is difficult to revise to a specific deadline. In the interest of protecting the conceptual advance provided by the work, and given the only minor revisions, we recommend a revision within 2 months (26th April 2026). Please discuss the revision progress ahead of this time with me if you require more time to complete the revisions.

I suggest that you publish the study as a short report, as it is formatted now. For short reports, the revised manuscript should not exceed 29,000 characters (including spaces but excluding materials & methods and references) and 5 main plus 5 expanded view figures. The results and discussion sections are combined. The "Conclusion and outlook" subheading needs to be deleted.

IMPORTANT NOTE: we perform an initial quality control of all revised manuscripts before re-review. Your manuscript will FAIL this control and the handling will be DELAYED if the following APPLIES:
1) A data availability section providing access to data deposited in public databases is missing. If you have not deposited any data, please add a sentence to the data availability section that explains that.
2) Your manuscript contains statistics and error bars based on n=2. Please use scatter blots in these cases. No statistics should be calculated if n=2.

When submitting your revised manuscript, please review the submission guidelines (https://link.springer.com/journal/44319/submission-guidelines#cms-Revised-submissions) and carefully review the instructions that follow below. Failure to include requested items will delay the evaluation of your revision.

1) a .docx formatted version of the manuscript text (including legends for main figures, EV figures and tables). Please make sure that the changes are highlighted to be clearly visible.

2) individual production quality figure files as .eps, .tif, .jpg (one file per figure). See hhttps://media.springernature.com/original/springer-cms/rest/v1/content/27825798/data/v1 for more info on how to prepare your figures.

3) We replaced Supplementary Information with Expanded View (EV) Figures and Tables that are collapsible/expandable online. A maximum of 5 EV Figures can be typeset. EV Figures should be cited as 'Figure EV1, Figure EV2" etc... in the text and their respective legends should be included in the main text after the legends of regular figures.

- For the figures that you do NOT wish to display as Expanded View figures, they should be bundled together with their legends in a single PDF file called *Appendix*, which should start with a short Table of Content. Appendix figures should be referred to in the main text as: "Appendix Figure S1, Appendix Figure S2" etc. See 'Expanded View' section of the submission guidelines.

- Additional Tables/Datasets should be labeled and referred to as Table EV1, Dataset EV1, etc. Legends have to be provided in a separate tab in case of .xls files. Alternatively, the legend can be supplied as a separate text file (README) and zipped together with the Table/Dataset file.

4) a .docx formatted letter INCLUDING the reviewers' reports and your detailed point-by-point responses to their comments. As part of the EMBO Press transparent editorial process, the point-by-point response is part of the Review Process File (RPF), which will be published alongside your paper.

5) a complete author checklist, which you can download from our author guidelines. Please insert information in the checklist that is also reflected in the manuscript. The completed author checklist will also be part of the RPF.

6) Please note that all corresponding authors are required to supply an ORCID ID for their name upon submission of a revised manuscript (), which can be linked to your profile in the manuscript submission system.

7) Before submitting your revision, primary datasets produced in this study need to be deposited in an appropriate public database. Please remember to provide a reviewer password if the datasets are not yet public. The accession numbers and database should be listed in a formal "Data Availability" section placed after Methods. Please note that the Data Availability Section is restricted to new primary data that are part of this study. * Note - All links should resolve to a page where the data can be accessed. *

If your study has not produced novel datasets, please mention this fact in the Data Availability Section.

8) At EMBO Press we ask authors to provide source data for the main manuscript figures. You will receive a separate email with instructions for providing source data with your revised manuscript, including how to upload and organize the files.

9) Our journal also encourages inclusion of *data citations in the reference list* to directly cite datasets that were re-used and obtained from public databases. Data citations in the article text are distinct from normal bibliographical citations and should directly link to the database records from which the data can be accessed. In the main text, data citations are formatted as follows: "Data ref: Smith et al, 2001" or "Data ref: NCBI Sequence Read Archive PRJNA342805, 2017". In the Reference list, data citations must be labeled with "[DATASET]". A data reference must provide the database name, accession number/identifiers and a resolvable link to the landing page from which the data can be accessed at the end of the reference.

10) Regarding data quantification, the following points must be specified in each figure legend:

- the name of the statistical test used to generate error bars and P values,

- the number (n) of independent experiments (please specify technical or biological replicates) underlying each data point,

- the nature of the bars and error bars (s.d., s.e.m.),

- If the data are obtained from n Program fragment delivered error ``Can't locate object method "less" via package "than" (perhaps you forgot to load "than"?) at //ejpvfs23/sites23b/embor_www/letters/embor_decision_revise_and_review_sr_any.txt line 55.' 2, use scatter blots showing the individual data points.

Discussion of statistical methodology can be reported in the materials and methods section, but figure legends should contain a basic description of n, P and the test applied.

- Please also include scale bars in all microscopy images.

11) The journal requires a statement specifying whether or not authors have competing interests (defined as all potential or actual interests that could be perceived to influence the presentation or interpretation of an article). In case of competing interests, this must be specified in your disclosure statement.

12) All Materials and Methods need to be described in the main text using our 'Structured Methods' format, which is required for all research articles. According to this format, the Methods section includes a separate Reagents and Tools Table file (listing key reagents, experimental models, software and relevant equipment and including their sources and relevant identifiers) and a Methods and Protocols section describing the methods using a step-by-step protocol format. The aim is to facilitate adoption of the methodologies across labs. More information on how to adhere to this format as well as a downloadable template (.docx) for the Reagents and Tools Table can be found in our author guidelines.

We would also welcome the submission of cover suggestions, or motifs to be used by our Graphics Illustrator in designing a cover.

As part of the EMBO publication's Transparent Editorial Process, EMBO reports publishes online a Review Process File (RPF) to accompany accepted manuscripts. This File will be published in conjunction with your paper and will include the referee reports, your point-by-point response and all pertinent correspondence relating to the manuscript.

You are able to opt out of this by letting the editorial office know (emboreports@embo.org). If you do opt out, the Review Process File link will point to the following statement: "No Review Process File is available with this article, as the authors have chosen not to make the review process public in this case."

I look forward to seeing a revised form of your manuscript when it is ready.

Best regards,
Esther

Esther Schnapp, PhD

Senior Editor
EMBO reports

Referee #1:

Cai et al report X-ray crystal structures and biochemical analysis of bacterial interstrand DAN crosslink glycosylase AlkX. Their structural and mutagenesis data support a DNA lesion sensing mechanism unique to this subclass of bacterial HTH_42 glycosylases. Overall, the data is of high quality, and the discussion is relevant and scholarly. I have some minor points for the authors consideration.

Points

1. Page. 7. The authors propose a non-base flipping mechanism "expected for an ICL glycosylase. Their mutagenesis data indicate a possible mechanism involves sensing of the crosslink by W124. An alternative is that crosslink induced distortions are sensed (or also sensed) via the minor groove opposite the lesion (e.g. via Y326)? Are there other possibilities to discuss regarding the expected mechanism of lesion detection?

2. E101 has no impact on catalysis. Did the authors try other residues in the vicinity of the THF - Q56, D58? On page 9 they state that they tested the QxD mutations in figure S4, but they are not found in that figure?

3. Page. 8. As written - the authors imply that the A89P mutation in Salmonella enterica confers tetracycline resistance, but then they discuss this further on page 9? For the sake of mechanism, I think the A89P data is relevant, but the discussion of the potential impacts on virulence seems confusing and tangential, at least for this referee.

4 Page.3 para3. Define "BGCs"

5. Overall the Figures are quite nice in general. Some suggestions for the authors consideration:

- Figure 4B-D. I would increase the size of these panels for clarity.
- Figure 2a. It is hard to see green type on black background.
- Some of the supplementary figures are low resolution, e.g. Figure S6A. S3-ABC

Referee #2:

DNA interstrand crosslinks (ICLs) are highly cytotoxic DNA lesions that covalently link two strands of DNA. Various DNA repair pathways act on ICLs, but until some years ago it was thought that DNA glycosylases that typically act by flipping out a nucleotide of the helix to excise a damage base would not be able to act on ICLs. This view changed by work primarily from the Eichman laboratory, which showed that some DNA glycosylases act by helix distortion rather than nucleotide flipping, allowing for the repair of ICL and other bulky lesions.

The structural and molecular mechanisms by which DNA glycosylases make incisions at ICLs remain poorly understood. To address this knowledge gap, this manuscript reports the structure of thermophilic AlkX, an ICL-processing DNA glycosylase that is for example found in the pathogenic bacterium A. baumannii, bound to the stable abasic site THF, representing the product of a reaction of AlkX with an ICL. The analysis of the structure clearly reveals how AlkX interacts with abasic sites through a DNA binding loop (BDL) from the minor groove and a loop with Trp124 in the major groove. Using biochemical assays with ICL substrates and cellular viability assays in A. baumannii, it is shown that the BDL (including Y300) and Trp124 are important for ICL repair activity. The authors further uncover an interaction network that supports the QxD active site motif, providing insight into how AlkX performs its catalytic activity.

Overall, this is well executed and clearly written manuscript that provides new structural and functional insights into the activity of DNA glycosylases on ICLs. The structures are explained well and supported by functional data that test the function of individual domains and residues. This manuscript is an excellent addition to EMBO Reports and I just have a few minor comments to be addressed prior to publication:

P3. Line 9 from bottom: Define BGCs (bacterial gene clusters)

Fig 1F: It would be helpful to include W124 in the scheme.

Fig 4C and accompanying text: The authors propose that W124 could be an "ICL sensor" based on the proximity to the NM ICL in the model. Could the authors elaborate on how they think this sensor operates? It could for example be that the aromatic ring

of the W interacts with the positively charged nitrogen in the NM ICL. A discussion of how this sensor might work would make the manuscript more interesting.

Referee #3:

The manuscript from Cai et al reports structural and biochemical studies of a bacterial DNA glycosylase that mediates repair of interstrand DNA crosslinks (ICLs), a highly toxic form of DNA damage. Their prior studies showed that the human pathogen A. baumannii has an ICL glycosylase, AlkX, but its mechanism for unhooking ICLs was unknown. Notably, unlike most DNA glycosylases, bacterial ICL glycosylases including AlkX and other members of the HTH_42 superfamily, do not use a nucleotide flipping, and knowledge of their mechanism for lesion recognition and repair is limited. They report a crystal structure of Thermobifida fusca AlkX (TfuAlkX) bound to DNA containing an abasic site (analog), representing a mimic of the product complex. Importantly, this is the first structure of a DNA-bound bacterial ICL glycosylase. TfuAlkX exhibits 30% AA sequence identity to A. baumannii AlkX and 34% identity to E. coli YcaQ. The work also identifies AlkX residues and motifs that mediate ICL repair, through in vitro activity assays and via sensitivity of A. baumannii to a crosslinking agent (mechlorethamine). Together, the structure and the follow-up studies to interrogate observed enzyme-DNA interactions, reveal conserved residues and motifs that mediate ICL unhooking.
The work provides an important advance in the understanding of how bacterial ICL glycosylases recognize and process ICLs in a mechanism that does not employ nucleotide flipping. I would suggest that the authors address the items listed below prior to publication.

1. It is stated in the Introduction that, "Genetic analysis showed that YcaQ initiates a secondary ICL repair pathway distinct from the known nucleotide excision ICL repair pathway." Does the latter pathway refer to the glycosylase activity of AlkX in unhooking ICLs? If so, it think it may be better to not refer to this as a "nucleotide excision" pathway since the AP site generated by AlkX is processed by BER.

2. The authors note that, "In addition to protecting cells against ICL agents, alkX is induced by, and protects against, acid stress,..." It could be informative to elaborate on this regarding the nature of the damage that is repaired by AlkX.

3. The abasic product used in the crystal structure (THF) is a stable mimic of a natural AP site. It would be informative to consider and discuss whether AlkX would potentially interact with the C1'-OH of a natural AP site.

4. When the authors first state that their new crystal structure of AlkX bound to DNA containing an abasic site represents the product of the ICL unhooking reaction. I think it should be noted that some elements of the actual product complex are missing, including the Gua-NM moiety.

5. On a related note - it would be helpful to note in the introduction how the remaining portion of the ICL is removed in these bacteria.

6. I think the flow could be improved if Fig 1A was shown as an independent figure, with the remaining panels in Fig 1 shown in a new figure in the Results section.

7. It might be better to avoid summarizing the results in the Introduction section; the significance, as written, is not clear without figures and relevant details. It may be better to just summarize the key questions addressed in the work.

8. In the results it is stated that, "...suggesting that AlkX does not remain bound to the excised nucleobase product." I'd suggest rewording to indicate that the base does not remain bound to the enzyme product complex. Also, might this observation might depend on the absence of an -OH at C1' of the THF analog? If so, perhaps this should be mentioned.

9. In the Results section it is noted that, "The THF (lesion) site is clamped between an extensive DNA-binding loop (DBL) from the β-sheet subdomain in the minor groove and a tryptophan side chain (W124) from the region between WH1 and WH2 in the major groove (Fig. 1D-F). Thus, the resolution and quality of the electron density in the structure reveal, for the first time, several critical interactions between a bacterial ICL glycosylase and damaged DNA." I think an additional figure that focuses on these specific interactions could help illustrate this key point. Figs 1D-F show the entire enzyme-DNA complex and don't clearly convey the interactions described above.

10. The authors note that, "...the structure suggests that the enzyme and/or DNA undergo a small conformational change after cleavage of the N-glycosidic bond, as observed in other alkylpurine DNA glycosylases (Metz et al, 2007)." I don't think it makes sense to refer to a glycosylase that uses base flipping as an example of the phenomenon that a conformational change occurs when an enzyme converts substrate to product. This is a common observation in enzyme catalysis and, in my opinion, does not need to be supported by an example, at least not one for an enzyme that exhibits a massive conformational change (base flipping) prior to the chemical step (unlike AlkX).

11. On a related note, could the possibility of a conformational change be supported by considering the model of AlkX bound to DNA with an NM-ICL shown in Fig 4C (the enzyme-substrate complex)?

12. Regarding the catalytic QxD residues, the authors conclude that in the current structure, "neither side chain (Q56 or D58) is close enough to the C1′ carbon to catalyze hydrolysis". Does the model of AlkX bound to DNA with an NM-ICL (Fig 4C) inform a potential role for D58 (or Q56) in binding/activating a water nucleophile? It seems that the orientation of the ribose sugar that contains the C1' electrophile is likely to differ substantially in the E-S vs E-P complexes.

13. In Fig 3A, it would be good to label positions of the THF ring (at least C1').

14. In my opinion, the paragraph on the catalytic (QxD) residues should have an appropriate section title, rather than falling under the heading "A genetic variant in S. enterica impairs ICL unhooking". Alternatively, the heading could potentially be modified.

15. While it is true that loss of DNA repair increases genetic variation and can contribute to adaptation, is there precedence for such a process triggered by defective ICL repair? If so, this should be stated in the paragraph discussing the effects of the A83P mutation and a potential role for this mutation, which hinders ICL repair, in contributing to tetracycline resistance.

16. In Fig 4A, it would help to use more distinct coloring for the Gua-NM-dG and the THF. In Fig 4C, the coloring used for the NM and AZB should be defined in the legend (pink, magenta, and orange moieties).

17. The observation that AlkX forms contacts with phosphates at the ends of the DNA used for crystallization raises the question of whether the activity of AlkX might be higher for a DNA that is longer than that used for crystallization.

Responses to reviewers
Cai, Kunkle et al

We thank the referees for taking their time to review our paper and for offering sound suggestions, which we feel have greatly improved the manuscript.

Referee #1:

Cai et al report X-ray crystal structures and biochemical analysis of bacterial interstrand DAN crosslink glycosylase AlkX. Their structural and mutagenesis data support a DNA lesion sensing mechanism unique to this subclass of bacterial HTH_42 glycosylases. Overall, the data is of high quality, and the discussion is relevant and scholarly. I have some minor points for the authors consideration.

Points

1. Page. 7. The authors propose a non-base flipping mechanism "expected for an ICL glycosylase. Their mutagenesis data indicate a possible mechanism involves sensing of the crosslink by W124. An alternative is that crosslink induced distortions are sensed (or also sensed) via the minor groove opposite the lesion (e.g. via Y326)? Are there other possibilities to discuss regarding the expected mechanism of lesion detection?

> Response: We agree that the DBL interaction with the minor groove plays a role in lesion detection. We have added additional discussion regarding ICL recognition throughout the text.

2. E101 has no impact on catalysis. Did the authors try other residues in the vicinity of the THF - Q56, D58? On page 9 they state that they tested the QxD mutations in figure S4, but they are not found in that figure?

> Response: We tested AbaAlkX QxD mutants Q39A and D41A (related to Q56 and D58 in TfuAlkX) in our previous work (Kunkle, Cai, et al, PMID: 38923984). The call-out to Fig. S4 in the sentence on p.9 referred to the DBL mutants. We have amended the text to clarify that the QxD mutants were tested previously.

3. Page. 8. As written - the authors imply that the A89P mutation in Salmonella enterica confers tetracycline resistance, but then they discuss this further on page 9? For the sake of mechanism, I think the A89P data is relevant, but the discussion of the potential impacts on virulence seems confusing and tangential, at least for this referee.

> Response: We agree with the reviewer and have removed this discussion paragraph. Although this pathogenic strain has accumulated an inactivating mutation in the AlkX homologue, the phenotypic impact(s) on the organism have not been explored.

4 Page.3 para3. Define "BGCs"

Response: We have now defined BGC on first use of “biosynthetic gene cluster” in the introduction.

5. Overall the Figures are quite nice in general. Some suggestions for the authors consideration:
- Figure 4B-D. I would increase the size of these panels for clarity.
- Figure 2a. It is hard to see green type on black background.
- Some of the supplementary figures are low resolution, e.g. Figure S6A. S3-ABC

Response: We have made these suggested changes to Figures 2A and 4, and have taken care to preserve resolution when converting the supplementary material to PDF.

Referee #2:

DNA interstrand crosslinks (ICLs) are highly cytotoxic DNA lesions that covalently link two strands of DNA. Various DNA repair pathways act on ICLs, but until some years ago it was thought that DNA glycosylases that typically act by flipping out a nucleotide of the helix to excise a damage base would not be able to act on ICLs. This view changed by work primarily from the Eichman laboratory, which showed that some DNA glycosylases act by helix distortion rather than nucleotide flipping, allowing for the repair of ICL and other bulky lesions.

The structural and molecular mechanisms by which DNA glycosylases make incisions at ICLs remain poorly understood. To address this knowledge gap, this manuscript reports the structure of thermophilic AlkX, an ICL-processing DNA glycosylase that is for example found in the pathogenic bacterium A. baumannii, bound to the stable abasic site THF, representing the product of a reaction of AlkX with an ICL. The analysis of the structure clearly reveals how AlkX interacts with abasic sites through a DNA binding loop (BDL) from the minor groove and a loop with Trp124 in the major groove. Using biochemical assays with ICL substrates and cellular viability assays in A. baumannii, it is shown that the BDL (including Y300) and Trp124 are important for ICL repair activity. The authors further uncover an interaction network that supports the QxD active site motif, providing insight into how AlkX performs its catalytic activity.

Overall, this is well executed and clearly written manuscript that provides new structural and functional insights into the activity of DNA glycosylases on ICLs. The structures are explained well and supported by functional data that test the function of individual domains and residues. This manuscript is an excellent addition to EMBO Reports and I just have a few minor comments to be addressed prior to publication:

P3. Line 9 from bottom: Define BGCs (bacterial gene clusters)

Response: We have defined BCG (biosynthetic gene cluster). Thank you for catching this error.

Fig 1F: It would be helpful to include W124 in the scheme.

Response: W124 does not make van der Waals or other contacts to the DNA, which is why it does not appear in the figure. However, we agree that it is useful to highlight where it is relative to the DNA, so we have revised the figure by adding its proximity to the DNA by a shaded region around the THF and the T8 nucleotide.

Fig 4C and accompanying text: The authors propose that W124 could be an "ICL sensor" based on the proximity to the NM ICL in the model. Could the authors elaborate on how they think this sensor operates? It could for example be that the aromatic ring of the W interacts with the positively charged nitrogen in the NM ICL. A discussion of how this sensor might work would make the manuscript more interesting.

Response: We think that the sensor operates by a direct interaction with the crosslink, either by van der Waals interaction or through a cation-π interaction as the reviewer suggests. We have added this to the main text.

Referee #3:

The manuscript from Cai et al reports structural and biochemical studies of a bacterial DNA glycosylase that mediates repair of interstrand DNA crosslinks (ICLs), a highly toxic form of DNA damage. Their prior studies showed that the human pathogen A. baumannii has an ICL glycosylase, AlkX, but its mechanism for unhooking ICLs was unknown. Notably, unlike most DNA glycosylases, bacterial ICL glycosylases including AlkX and other members of the HTH_42 superfamily, do not use a nucleotide flipping, and knowledge of their mechanism for lesion recognition and repair is limited. They report a crystal structure of Thermobifida fusca AlkX (TfuAlkX) bound to DNA containing an abasic site (analog), representing a mimic of the product complex. Importantly, this is the first structure of a DNA-bound bacterial ICL glycosylase. TfuAlkX exhibits 30% AA sequence identity to A. baumannii AlkX and 34% identity to E. coli YcaQ. The work also identifies AlkX residues and motifs that mediate ICL repair, through in vitro activity assays and via sensitivity of A. baumannii to a crosslinking agent (mechlorethamine). Together, the structure and the follow-up studies to interrogate observed enzyme-DNA interactions, reveal conserved residues and motifs that mediate ICL unhooking.
The work provides an important advance in the understanding of how bacterial ICL glycosylases recognize and process ICLs in a mechanism that does not employ

nucleotide flipping. I would suggest that the authors address the items listed below prior to publication.

1. It is stated in the Introduction that, "Genetic analysis showed that YcaQ initiates a secondary ICL repair pathway distinct from the known nucleotide excision ICL repair pathway." Does the latter pathway refer to the glycosylase activity of AlkX in unhooking ICLs? If so, it think it may be better to not refer to this as a "nucleotide excision" pathway since the AP site generated by AlkX is processed by BER.

Response: No, the latter pathway refers to the UvrA-mediated nucleotide excision ICL repair pathway. We have revised the text to clarify.

2. The authors note that, "In addition to protecting cells against ICL agents, alkX is induced by, and protects against, acid stress,..." It could be informative to elaborate on this regarding the nature of the damage that is repaired by AlkX.

Response: We know that acid stress, like that encountered during interactions with the host immune system, induce broad DNA damage. The exact nature of the adducts produced in this environment are unknown, although they are likely multifaceted. Given AlkX's specificity, the fact that its loss from cells show increased sensitivity to acid suggests that ICL's are included in that repertoire, although directly identifying individual DNA lesions is highly difficult and has not been done. Therefore, we have added text to state that the relationship between the acid stress and DNA damage responses are unclear.

3. The abasic product used in the crystal structure (THF) is a stable mimic of a natural AP site. It would be informative to consider and discuss whether AlkX would potentially interact with the C1'-OH of a natural AP site.

Response: We have certainly considered this, but there simply are no interactions with the C1' carbon in the current structure. Thus, it is difficult to speculate if AlkX would interact with a natural abasic site any differently than what we observe here. The lack of an interaction with C1' is described in the first paragraph of the active site section of the results.

4. When the authors first state that their new crystal structure of AlkX bound to DNA containing an abasic site represents the product of the ICL unhooking reaction. I think it should be noted that some elements of the actual product complex are missing, including the Gua-NM moiety.

Response: We have clarified that the AP-site is one-half of the product at the end of the Introduction, and we now explicitly state that the structure does not include the Gua-NM-dG monoadduct at the start of the Results.

5. On a related note - it would be helpful to note in the introduction how the remaining portion of the ICL is removed in these bacteria.

Response: We have added what we know about how the product of the AlkX unhooking product is processed to the Introduction. In short, our previous genetic analysis of mechlorethamine sensitivity to *ΔycaQ* cells is strongly dependent on the AP endonuclease EndoIV, and thus we believe that both the AP site and remaining NM-monoadduct are processed via BER (Bradley, et al, PMID: 32409837).

6. I think the flow could be improved if Fig 1A was shown as an independent figure, with the remaining panels in Fig 1 shown in a new figure in the Results section.

Response: We appreciate this idea, but we would prefer to keep Fig 1A together with the structure since there are elements of the reaction scheme (e.g., structure of THF, position of crosslinked nucleotides) that are more convenient to show with the structure and Fig. 1E.

7. It might be better to avoid summarizing the results in the Introduction section; the significance, as written, is not clear without figures and relevant details. It may be better to just summarize the key questions addressed in the work.

Response: We have edited the last paragraph of the introduction to more clearly articulate the key questions and to remove some of the details of the results. However, we kept the results summary since we are limited by what can be summarized in the abstract because of space limitations.

8. In the results it is stated that, "...suggesting that AlkX does not remain bound to the excised nucleobase product." I'd suggest rewording to indicate that the base does not remain bound to the enzyme product complex. Also, might this observation might depend on the absence of an -OH at C1' of the THF analog? If so, perhaps this should be mentioned.

Response: We have reworded the sentence accordingly. We do not think that this observation depends on our use of a THF analog since we have trapped structures containing THF-DNA and 7mGua previously (e.g., Mullins et al, PMID 26524531).

9. In the Results section it is noted that, "The THF (lesion) site is clamped between an extensive DNA-binding loop (DBL) from the β-sheet subdomain in the minor groove and a tryptophan side chain (W124) from the region between WH1 and WH2 in the major groove (Fig. 1D-F). Thus, the resolution and quality of the electron density in the structure reveal, for the first time, several critical interactions between a bacterial ICL glycosylase and damaged DNA." I think an additional figure that focuses on these specific interactions could help illustrate this key point. Figs 1D-F show the entire enzyme-DNA complex and don't clearly convey the interactions described above.

Response: The specific interactions are shown in Figures 2-4. We have altered the sentence to read “Thus, the resolution and quality of the electron density in the structure reveal, for the first time, several critical interactions between a bacterial ICL glycosylase and damaged DNA *that we describe in more detail below*.”

10. The authors note that, "...the structure suggests that the enzyme and/or DNA undergo a small conformational change after cleavage of the N-glycosidic bond, as observed in other alkylpurine DNA glycosylases (Metz et al, 2007)." I don't think it makes sense to refer to a glycosylase that uses base flipping as an example of the phenomenon that a conformational change occurs when an enzyme converts substrate to product. This is a common observation in enzyme catalysis and, in my opinion, does not need to be supported by an example, at least not one for an enzyme that exhibits a massive conformational change (base flipping) prior to the chemical step (unlike AlkX).

Response: We have removed “as observed in other alkylpurine DNA glycosylases (Metz et al, 2007).”

11. On a related note, could the possibility of a conformational change be supported by considering the model of AlkX bound to DNA with an NM-ICL shown in Fig 4C (the enzyme-substrate complex)?

Response: We do not believe that the NM-ICL model shown in Fig 4C should be used to support this suggestion of a conformation change. The model in Fig 4C is a product complex that lacks the constraint that would exist in the ICL substrate, and was made simply by adding a Gua-NM-dG moiety to show the proximity of W124 to the crosslink. We believe the structure as shown in Fig 3A makes the point regarding the fact that the DNA product is not in a catalytic configuration.

12. Regarding the catalytic QxD residues, the authors conclude that in the current structure, "neither side chain (Q56 or D58) is close enough to the C1′ carbon to catalyze hydrolysis". Does the model of AlkX bound to DNA with an NM-ICL (Fig 4C) inform a potential role for D58 (or Q56) in binding/activating a water nucleophile? It seems that the orientation of the ribose sugar that contains the C1' electrophile is likely to differ substantially in the E-S vs E-P complexes.

Response: Please see our response to point #11.

13. In Fig 3A, it would be good to label positions of the THF ring (at least C1').

Response: We have labeled C1′ in Fig 3A.

14. In my opinion, the paragraph on the catalytic (QxD) residues should have an appropriate section title, rather than falling under the heading "A genetic variant in S. enterica impairs ICL unhooking". Alternatively, the heading could potentially be modified.

Response: We have retitled the section to “The AlkX active site contains and a genetic variant in *S. enterica* that impairs ICL repair.”

15. While it is true that loss of DNA repair increases genetic variation and can contribute to adaptation, is there precedence for such a process triggered by defective ICL repair? If so, this should be stated in the paragraph discussing the effects of the A83P mutation and a potential role for this mutation, which hinders ICL repair, in contributing to tetracycline resistance.

Response: This is an excellent question. We are unaware of studies showing that impaired ICL repair leads to genetic variation and resistance, and therefore have decided to remove this discussion paragraph.

16. In Fig 4A, it would help to use more distinct coloring for the Gua-NM-dG and the THF. In Fig 4C, the coloring used for the NM and AZB should be defined in the legend (pink, magenta, and orange moieties).

Response: We have lightened the pink color of the Gua-NM-dG in Fig 4A and have revised the legend for Fig 4C.

17. The observation that AlkX forms contacts with phosphates at the ends of the DNA used for crystallization raises the question of whether the activity of AlkX might be higher for a DNA that is longer than that used for crystallization.

Response: We absolutely agree. Whereas an 18-base pair DNA was used for crystallization, the DNA substrates used for biochemical activity measurements were 26 base pairs in length.

Dear Dr. Eichman,

Thank you for the submission of your revised manuscript. It looks all good now and only some editorial requests will need to be addressed before we can proceed with the official acceptance of your manuscript:

- The author credits need to be removed from the ms file. All credits are entered during online ms submission.

- The uploaded Dataset EV1 should be part of the Reagents & Tools table. Or, if you prefer, it could be called Table EV2 and cited in the R&T table. The R&T table needs to removed from the ms file, we only need it as separate file.

- The APPENDIX FILE is missing a table of content with page numbers on the first page. Also, the nomenclature in the file is incorrect, it needs to be Appendix Figure S1, etc., please correct.

* Figure Legends - Comments *

- Please note that the exact p values are not provided in the legends of figures 2E, 3F, please provide exact values as reasonable.
- Please note that information related to n is missing in the legends of figures EV4 A-F
- Please note that the error bars are not defined in the legends of figures EV4 A-F.

I slightly modified your abstract that needs to be written in present tense when it comes to the description of your findings:

Here, we describe a crystal structure of AlkX bound to DNA representing a product of the ICL unhooking reaction. Mutational analysis of ICL unhooking in vitro and A. baumannii sensitivity to the crosslinking agent mechlorethamine enable the identification of several AlkX motifs critical for ICL repair. We also find that a genetic variant from an antibiotic-resistant strain of the human pathogen Salmonella enterica reduces AlkX activity in vitro and increases A. baumannii sensitivity to DNA crosslinking. This work provides a structural basis for how bacterial ICL glycosylases recognize and repair DNA adducts and contributes additional evidence that ICL repair is important for fitness of human pathogens.

EMBO press papers are accompanied online by A) a short (1-2 sentences) summary of the findings and their significance, B) 2-3 bullet points highlighting key results and C) a synopsis image that is exactly 550 pixels wide and 200-600 pixels high (the height is variable). The synopsis image should provide a sketch of the major findings, like a graphical abstract. Please note that text needs to be readable at the final size. Please send us this information along with the final manuscript.

I look forward to seeing a final version of your manuscript as soon as possible.

You should be able to bring forward all ms files from this version of the ms to the new V3 version and you can then replace only the files that need to be replaced.

Best regards,
Esther

Esther Schnapp, PhD
Senior Editor
EMBO reports

The authors have addressed all minor editorial requests.

Dr. Brandt Eichman
Vanderbilt University
Biological Sciences
465 21st Ave S
MRBIII 5270A
Nashville, TN 37232
United States

Dear Dr. Eichman,

I am very pleased to accept your manuscript for publication in the next available issue of EMBO reports. Thank you for your contribution to our journal.

Your manuscript will be processed for publication by EMBO Press. It will be copy edited and you will receive page proofs prior to publication. Please note that you will be contacted by Springer Nature Author Services to complete licensing and payment information.

You may qualify for financial assistance for your publication charges - either via a Springer Nature fully open access agreement or an EMBO initiative. Check your eligibility: https://link.springer.com/journal/44319/how-to-publish-with-us

Should you be planning a Press Release on your article, please get in contact with embo_production@springernature.com as early as possible in order to coordinate publication and release dates.

If you have any questions, please do not hesitate to contact the Editorial Office. Thank you for your contribution to EMBO Reports.

Best regards,
Esther

Esther Schnapp, PhD
Senior Editor
EMBO reports

>>> Please note that it is EMBO Reports policy for the transcript of the editorial process (containing referee reports and your response letter) to be published as an online supplement to each paper. If you do NOT want this, you will need to inform the Editorial Office via email immediately. More information is available here: https://link.springer.com/partners/embo-press/editorial-policies#Peer%20review

EMBO Press Author Checklist

Corresponding Author Name: Brandt F. Eichman and Eric P. Skaar	
Journal Submitted to: EMBO Reports	
Manuscript Number: EMBOR-2025-63381V1	

USEFUL LINKS FOR COMPLETING THIS FORM
The EMBO Journal - Author Guidelines
EMBO Reports - Author Guidelines
Molecular Systems Biology - Author Guidelines
EMBO Molecular Medicine - Author Guidelines

Reporting Checklist for Life Science Articles (updated January
This checklist is adapted from Materials Design Analysis Reporting (MDAR) Checklist for Authors. MDAR establishes a minimum set of requirements in transparent reporting in the life sciences (see Statement of Task: 10.31222/osf.io/9sm4x). Please follow the journal's guidelines in preparing your
Please note that a copy of this checklist will be published alongside your article.

Abridged guidelines for figures
1. Data
The data shown in figures should satisfy the following conditions:
- the data were obtained and processed according to the field's best practice and are presented to reflect the results of the experiments in an accurate and unbiased manner.
- ideally, figure panels should include only measurements that are directly comparable to each other and obtained with the same assay.
- plots include clearly labeled error bars for independent experiments and sample sizes. Unless justified, error bars should not be shown for technical
- if n<5, the individual data points from each experiment should be plotted. Any statistical test employed should be justified.
- Source Data should be included to report the data underlying figures according to the guidelines set out in the authorship guidelines on Data

2. Captions
Each figure caption should contain the following information, for each panel where they are relevant:
- a specification of the experimental system investigated (eg cell line, species name).
- the assay(s) and method(s) used to carry out the reported observations and measurements.
- an explicit mention of the biological and chemical entity(ies) that are being measured.
- an explicit mention of the biological and chemical entity(ies) that are altered/varied/perturbed in a controlled manner.
- the exact sample size (n) for each experimental group/condition, given as a number, not a range;
- a description of the sample collection allowing the reader to understand whether the samples represent technical or biological replicates (including how many animals, litters, cultures, etc.).
- a statement of how many times the experiment shown was independently replicated in the laboratory.
- definitions of statistical methods and measures:
  - common tests, such as t-test (please specify whether paired vs. unpaired), simple χ2 tests, Wilcoxon and Mann-Whitney tests, can be unambiguously identified by name only, but more complex techniques should be described in the methods section;
  - are tests one-sided or two-sided?
  - are there adjustments for multiple comparisons?
  - exact statistical test results, e.g., P values = x but not P values < x;
  - definition of 'center values' as median or average;
  - definition of error bars as s.d. or s.e.m.

Please complete ALL of the questions below.
Select "Not Applicable" only when the requested information is not relevant for your study.

Materials

Newly Created Materials	Information included in the manuscript?	In which section is the information available? (Reagents and Tools Table, Materials and Methods, Figures, Data Availability Section)
New materials and reagents need to be available; do any restrictions apply?	Yes	Reagents and Tools Table

Antibodies	Information included in the manuscript?	In which section is the information available? (Reagents and Tools Table, Materials and Methods, Figures, Data Availability Section)
For **antibodies** provide the following information:<br>- Commercial antibodies: RRID (if possible) or supplier name, catalogue number and or/clone number<br>- Non-commercial: RRID or citation	Not Applicable	

DNA and RNA sequences	Information included in the manuscript?	In which section is the information available? (Reagents and Tools Table, Materials and Methods, Figures, Data Availability Section)
**Short novel DNA or RNA including primers, probes**: provide the sequences.	Yes	Reagents and Tools Table

Cell materials	Information included in the manuscript?	In which section is the information available? (Reagents and Tools Table, Materials and Methods, Figures, Data Availability Section)
**Cell lines:** Provide species information, strain. Provide accession number in repository **OR** supplier name, catalog number, clone number, and/**OR** RRID.	Not Applicable	
**Primary cultures:** Provide species, strain, sex of origin, genetic modification status.	Not Applicable	
Report if the cell lines were recently **authenticated** (e.g., by STR profiling) and tested for mycoplasma contamination.	Not Applicable	

Experimental animals	Information included in the manuscript?	In which section is the information available? (Reagents and Tools Table, Materials and Methods, Figures, Data Availability Section)
**Laboratory animals or Model organisms:** Provide species, strain, sex, age, genetic modification status. Provide accession number in repository **OR** supplier name, catalog number, clone number, **OR** RRID.	Not Applicable	
**Animal observed in or captured from the field:** Provide species, sex, and age where possible.	Not Applicable	
Please detail **housing and husbandry conditions**.	Not Applicable	

Plants and microbes	Information included in the manuscript?	In which section is the information available? (Reagents and Tools Table, Materials and Methods, Figures, Data Availability Section)
**Plants:** provide species and strain, ecotype and cultivar where relevant, unique accession number if available, and source (including location for collected wild specimens).	Not Applicable	
**Microbes:** provide species and strain, unique accession number if available, and source.	Yes	Reagents and Tools Table

Human research participants	Information included in the manuscript?	In which section is the information available? (Reagents and Tools Table, Materials and Methods, Figures, Data Availability Section)
If collected and within the bounds of privacy constraints report on age, sex and gender or ethnicity for all study participants.	Not Applicable	

Core facilities	Information included in the manuscript?	In which section is the information available? (Reagents and Tools Table, Materials and Methods, Figures, Data Availability Section)
If your work benefited from core facilities, was their service mentioned in the acknowledgments section?	Yes	Materials and Methods

Design

Study protocol	Information included in the manuscript?	In which section is the information available? (Reagents and Tools Table, Materials and Methods, Figures, Data Availability Section)
If study protocol has been **pre-registered, provide DOI in the manuscript**. For clinical trials, provide the trial registration number **OR** cite DOI.	Not Applicable	
Report the **clinical trial registration number** (at ClinicalTrials.gov or equivalent), where applicable.	Not Applicable	

Laboratory protocol	Information included in the manuscript?	In which section is the information available? (Reagents and Tools Table, Materials and Methods, Figures, Data Availability Section)
Provide DOI OR other citation details if **external detailed step-by-step protocols** are available.	Not Applicable	

Experimental study design and statistics	Information included in the manuscript?	In which section is the information available? (Reagents and Tools Table, Materials and Methods, Figures, Data Availability Section)
Include a statement about **sample size** estimate even if no statistical methods were used.	Yes	Figures
Were any steps taken to minimize the effects of subjective bias when allocating animals/samples to treatment (e.g. **randomization procedure**)? If yes, have they been described?	Not Applicable	
Include a statement about **blinding** even if no blinding was done.	Not Applicable	
Describe **inclusion/exclusion criteria** if samples or animals were excluded from the analysis. Were the criteria pre-established?<br><br>If sample or data points were omitted from analysis, report if this was due to attrition or intentional exclusion and provide justification.	Not Applicable	
For every figure, are **statistical tests** justified as appropriate? Do the data meet the assumptions of the tests (e.g., normal distribution)? Describe any methods used to assess it. Is there an estimate of variation within each group of data? Is the variance similar between the groups that are being statistically compared?	Yes	Figures

Sample definition and in-laboratory replication	Information included in the manuscript?	In which section is the information available? (Reagents and Tools Table, Materials and Methods, Figures, Data Availability Section)
In the figure legends: state number of times the experiment was **replicated** in laboratory.	Yes	Figures
In the figure legends: define whether data describe **technical or biological replicates**.	Yes	Figures

Ethics

Ethics	Information included in the manuscript?	In which section is the information available? (Reagents and Tools Table, Materials and Methods, Figures, Data Availability Section)
Studies involving **human participants**: State details of **authority granting ethics approval** (IRB or equivalent committee(s), provide reference number for approval.	Not Applicable	
Studies involving **human participants**: Include a statement confirming that **informed consent** was obtained from all subjects and that the experiments conformed to the principles set out in the WMA Declaration of Helsinki and the Department of Health and Human Services Belmont Report.	Not Applicable	
Studies involving **human participants:** For publication of **patient photos**, include a statement confirming that consent to publish was obtained.	Not Applicable	
Studies involving experimental **animals**: State details of **authority granting ethics approval** (IRB or equivalent committee(s), provide reference number for approval. Include a statement of compliance with ethical regulations.	Not Applicable	
Studies involving **specimen and field samples:** State if relevant **permits** obtained, provide details of authority approving study; if none were required, explain why.	Not Applicable	

Dual Use Research of Concern (DURC)	Information included in the manuscript?	In which section is the information available? (Reagents and Tools Table, Materials and Methods, Figures, Data Availability Section)
Could your study fall under dual use research restrictions? Please check biosecurity documents and list of **select agents and toxins** (CDC): https://www.selectagents.gov/sat/list.htm	Not Applicable	
If you used a select agent, is the security level of the lab appropriate and reported in the manuscript?	Not Applicable	
If a study is subject to dual use research of concern regulations, is the name of the **authority granting approval and reference number** for the regulatory approval provided in the manuscript?	Not Applicable	

Reporting

The MDAR framework recommends adoption of discipline-specific guidelines, established and endorsed through community initiatives. Journals have their own policy about requiring specific guidelines and recommendations to complement MDAR.

Adherence to community standards	Information included in the manuscript?	In which section is the information available? (Reagents and Tools Table, Materials and Methods, Figures, Data Availability Section)
State if relevant guidelines or checklists (e.g., **ICMJE, MIBBI, ARRIVE, PRISMA**) have been followed or provided.	Not Applicable	
For **tumor marker prognostic studies,** we recommend that you follow the **REMARK** reporting guidelines (see link list at top right). See author guidelines, under 'Reporting Guidelines'. Please confirm you have followed these guidelines.	Not Applicable	
For **phase II and III randomized controlled trials**, please refer to the **CONSORT** flow diagram (see link list at top right) and submit the CONSORT checklist (see link list at top right) with your submission. See author guidelines, under 'Reporting Guidelines'. Please confirm you have submitted this list.	Not Applicable	

Data Availability

Data availability	Information included in the manuscript?	In which section is the information available? (Reagents and Tools Table, Materials and Methods, Figures, Data Availability Section)
Have **primary datasets** been deposited according to the journal's guidelines (see 'Data Deposition' section) and the respective accession numbers provided in the Data Availability Section?	Yes	Data Availability Section
Were **human clinical and genomic datasets** deposited in a public access-controlled repository in accordance to ethical obligations to the patients and to the applicable consent agreement?	Not Applicable	
Are **computational models** that are central and integral to a study available without restrictions in a machine-readable form? Were the relevant accession numbers or links provided?	Not Applicable	
If publicly available data were reused, provide the respective **data citations in the reference list**.	Not Applicable	
